# Synergistic Antibacterial and Antioxidant Activities of Folium *Aloe*–Cortex *Betulae* Drug Combination: Optimization via Box–Behnken Design and In Vitro Validation

**DOI:** 10.3390/molecules31152590

**Published:** 2026-07-24

**Authors:** Shun Zhang, Kun Bao, Yiyun Wei, Xu Liu, Chenlu Zhang

**Affiliations:** 1 School of Biological Science and Engineering, Shaanxi University of Technology, Hanzhong 723000, China; 15929874564@163.com (S.Z.); bkc0822@163.com (K.B.); 17764778099@163.com (Y.W.); 15667909234@163.com (X.L.); 2 Shaanxi Province Key Laboratory of Bio-Resources, Hanzhong 723000, China; 3 Qinba State Key Laboratory of Biological Resources and Ecological Environment (Incubation), Hanzhong 723000, China

**Keywords:** Folium *Aloe*, Cortex *Betulae*, UPLC-QTOF-MS, response surface methodology, Box–Behnken design

## Abstract

This study aimed to screen for optimal Traditional Chinese medicine (TCM) combinations and subsequently optimize their extraction processes for developing antibacterial dressings by preparing aqueous and hydroethanolic extracts from Herba *Centellae asiaticae*, Flos *Calendulae*, Folium *Aloe*, Cortex *Betulae* and Rhizoma *Curcumae longae* and evaluating their inhibitory activity against *E. coli*, *S. aureus*, *C. albicans*, and *T-Salmonella* using an agar diffusion assay. Synergistic combinations were identified through a grid screening method, with extraction processes optimized via single-factor experiments and Box–Behnken response surface design. The results demonstrated that the Folium *Aloe* and Cortex *Betulae* formulation exhibited the strongest synergistic antibacterial activity, with the inhibition zone expanding from 11.1/12.8 mm for individual components to 17 mm after combination, representing an increase of 21–36%. Single-factor and response surface optimization identified optimal process parameters as a ratio of 1.53:1, a hydroethanolic concentration of 79.5%, and an extraction time of 2.6 h, with a model R^2^ value ranging from 0.9265 to 0.9783; the MIC values were reduced by 25–68.75%. The UPLC-QTOF-MS analysis of the optimized extract identified nine major constituents, including anthraquinones, chromones, and triterpenoids, providing the phytochemical basis for the observed bioactivities. Mechanistic studies via UV-Vis spectrophotometry and propidium iodide (PI) staining confirmed membrane disruption through nucleic acid and protein leakage. Cytocompatibility evaluation using L929 fibroblasts demonstrated >90% cell viability even at 1000 μg/mL. Additionally, the optimized extract exhibited concentration-dependent antibacterial activity, achieving a DPPH scavenging rate of 90% at 1.0 mg/mL, whereas its ABTS scavenging rate was comparable to that of ascorbic acid. The Folium *Aloe* and Cortex *Betulae* pair demonstrates dual antibacterial and antioxidant activities, providing an experimental basis for developing functional wound dressings and green antibacterial biomaterials.

## 1. Introduction

Chronic wound infections represent a significant clinical challenge worldwide, particularly among patients with diabetes, burns, or immunocompromised conditions [1,2]. These wounds are often colonized by diverse microbial communities, including Gram-positive bacteria, Gram-negative bacteria, and fungi, which interact synergistically to exacerbate the inflammatory response and delay healing [3,4]. Crucially, chronic wound infections are characterized by a pathological feedback loop: polymicrobial colonization (*S. aureus*, *E. coli*, *Pseudomonas aeruginosa*, *C. albicans*) triggers excessive reactive oxygen species (ROS) production, which in turn impairs immune cell function, degrades the extracellular matrix, and creates a pro-inflammatory microenvironment that sustains infection [5,6]. Natural medicines, particularly traditional Chinese medicine, exhibit broad application prospects due to their multi-component and multi-target characteristics that align closely with the complex pathology of chronic wounds [7,8,9]. Folium *Aloe* is rich in polysaccharides and anthraquinone compounds, demonstrating moisturizing effects, promoting cell proliferation, and exhibiting broad-spectrum antibacterial activity [9,10]. Cortex *Betulae* is rich in triterpenoid compounds such as birch alcohol, which exhibit selective inhibitory effects against Gram-positive bacteria and potent anti-inflammatory activity [11,12]; Herba Flos *Calendulae* carotenoids and flavonoids accelerate inflammation resolution [13]; Herba *Centellae asiaticae* triterpenoid saponins promote collagen synthesis and angiogenesis [14]; and *Curcumae longae* demonstrates strong antioxidant and anti-resistant bacterial activity [15]. However, significant differences exist among these five traditional Chinese medicines in terms of reducing activity, antibacterial spectrum profiles, and synthetic stability, necessitating the application of rigorous screening methods to determine the optimal combination.

The convergence of antibacterial and antioxidant mechanisms in these herbal raw materials is chemically grounded: polyphenolic compounds can both disrupt bacterial membranes via redox cycling and scavenge free radicals via hydrogen donation, where extract constituents simultaneously attack pathogens and resolve oxidative stress—mimicking the integrated therapeutic logic of TCM herbal pairs [9,16,17]. The pairing of Folium *Aloe* and Cortex *Betulae* is supported by solid scientific evidence: Folium *Aloe* polysaccharides enhance the membrane permeability of birch alcohol, facilitating its transmembrane transport; birch alcohol stabilizes the anthraquinone components in Folium *Aloe* and delays oxidative degradation. Folium *Aloe* exhibits significant efficacy against Gram-negative bacteria and fungi, whereas Cortex *Betulae* demonstrates pronounced selective inhibition of Gram-positive bacteria, resulting in complementary antimicrobial spectra. Folium *Aloe* promotes cell proliferation and moisturization, whereas Cortex *Betulae* exhibits anti-inflammatory and angiogenic effects, collectively forming a repair–inflammation synergy that encompasses the entire wound healing process [5,9,10,18]. However, the synergistic effects of these pairs require quantitative validation, and their extraction processes need optimization to maximize the yield of active constituents.

This study establishes a systematic “screening–combination–optimization–validation” framework. Specifically, five herbal raw materials were first evaluated via dual-solvent extraction and agar diffusion against four wound pathogens to identify candidates with broad antibacterial activity. The optimal pair was then determined through grid-based compatibility screening, followed by single-factor and Box–Behnken response surface optimization of extraction parameters. Finally, the optimized extract was comprehensively characterized by UPLC-QTOF-MS for phytochemical profiling, and its antibacterial mechanism, antioxidant capacity, and cytocompatibility were validated through a series of in vitro assays, providing a replicable paradigm for TCM-based wound dressing development.

## 2. Results and Discussion

### 2.1. Screening of Single Medicinal Herbs

**Figure 1:**
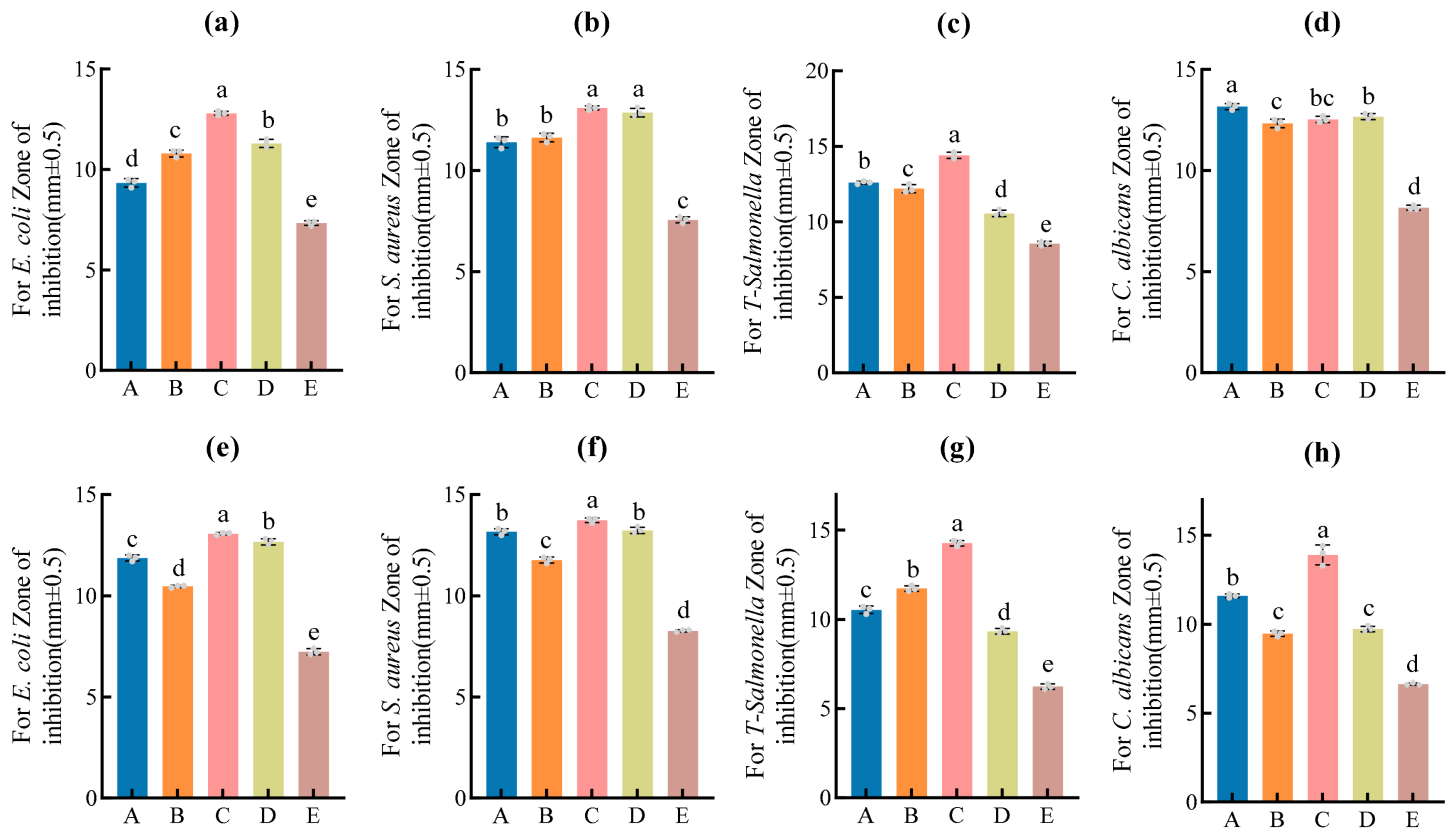
The inhibition profiles of traditional Chinese medicine extracts against four bacterial strains, (**a**–**d**) aqueous extracts; (**e**–**h**) hydroethanolic extracts; A–E. Herba *Centellae asiaticae*, Flos *Calendulae*, Folium *Aloe*, Cortex *Betulae* and Rhizoma *Curcumae longae*. The letters a, b, c, d and e represent different levels of statistical significance.

This study selected five traditional Chinese medicines with documented wound-healing and antibacterial activities: Herba *Centellae asiaticae*, Flos *Calendulae*, Folium *Aloe*, Cortex *Betulae* and Rhizoma *Curcumae longae*. However, significant differences were observed in their reducing activity, antibacterial spectrum profiles, and extraction stability. The agar well diffusion method was employed to evaluate the antibacterial efficacy of aqueous extracts and 75% hydroethanolic extracts from these five herbs against four representative wound pathogens (Supplementary Figure S1). The results in Figure 1 demonstrate that each herb exhibits distinct antibacterial spectra and solvent-dependent activity profiles.

The water extraction results (Figure 1a–d) demonstrate that Cortex *Betulae* exhibits the most potent inhibitory effects against *S. aureus*, *E. coli*, and *T-Salmonella*, likely attributed to the disruptive action of its high concentrations of birch alcohol and phenolic compounds on bacterial membrane integrity [18]. Folium *Aloe* water extract showed significant activity against *S. aureus*, whereas Herba *Centellae asiaticae* exhibited selective inhibitory effects against *C. albicans*. These findings indicate that water extraction better preserves polar antibacterial components, which exhibit excellent solubility and biological activity in aqueous environments. The 75% hydroethanolic extract results revealed distinct antimicrobial characteristics (Figure 1e–h). Cortex *Betulae* exhibited superior inhibitory activity against all four test strains, consistent with the broad-spectrum efficacy of its lipophilic triterpenoids and phenolic compounds in hydroethanolic [19]. Notably, Folium *Aloe* hydroethanolic extract demonstrated superior inhibitory effects against all four pathogenic bacteria compared to its aqueous extract, indicating that hydroethanolic is more effective in extracting lipophilic antibacterial components from Folium *Aloe* [9]. This solvent-dependent difference underscores the critical role of extraction methods in determining the antimicrobial potential of herbal extracts. In contrast, Flos *Calendulae* and Cortex *Betulae* exhibited stronger activity in the aqueous extracts of specific strains, suggesting that their primary polar bioactive components are better preserved under aqueous conditions.

Rhizoma *Curcumae longae* consistently demonstrated the poorest efficacy across all eight experimental groups (two extraction methods × four pathogenic bacteria), with a significantly smaller inhibition zone diameter compared to other extracts (*p* < 0.01). This suboptimal performance may stem from curcumin’s low water solubility and thermal instability, which lead to its susceptibility to degradation during extraction and consequently diminish its antibacterial potency. These findings align with the limitations of curcumin-based antimicrobial preparations reported by Chang et al. [20]. Consequently, turmeric was excluded from subsequent drug screening studies.

A comprehensive analysis of the two extraction systems identified Herba *Centellae asiaticae*, Flos *Calendulae*, Folium *Aloe* and Cortex *Betulae* as candidate herbal raw materials with definitive antibacterial activity. Based on these findings, the four active compounds were subjected to pairwise compatibility screening to verify whether this complementarity could translate into a synergistic enhancement effect.

### 2.2. Screening of Drug Combination Pairs

Based on the screening results of individual herbs, four herbal raw materials with confirmed antibacterial activity were selected for pairwise combination testing. Six herb pair combinations were established: Herba *Centellae asiaticae* + Cortex *Betulae*, Flos *Calendulae* + Cortex *Betulae*, Folium *Aloe* + Cortex *Betulae*, Flos *Calendulae* + Folium *Aloe*, Flos *Calendulae* + Folium *Aloe*, and Flos *Calendulae* + Flos *Calendulae*. Each combination was prepared using the grid method at a 1:1 mass ratio, and both aqueous extracts and hydroethanolic extracts were evaluated for their antibacterial activity against the four test bacterial strains (Supplementary Figure S2).

**Figure 2:**
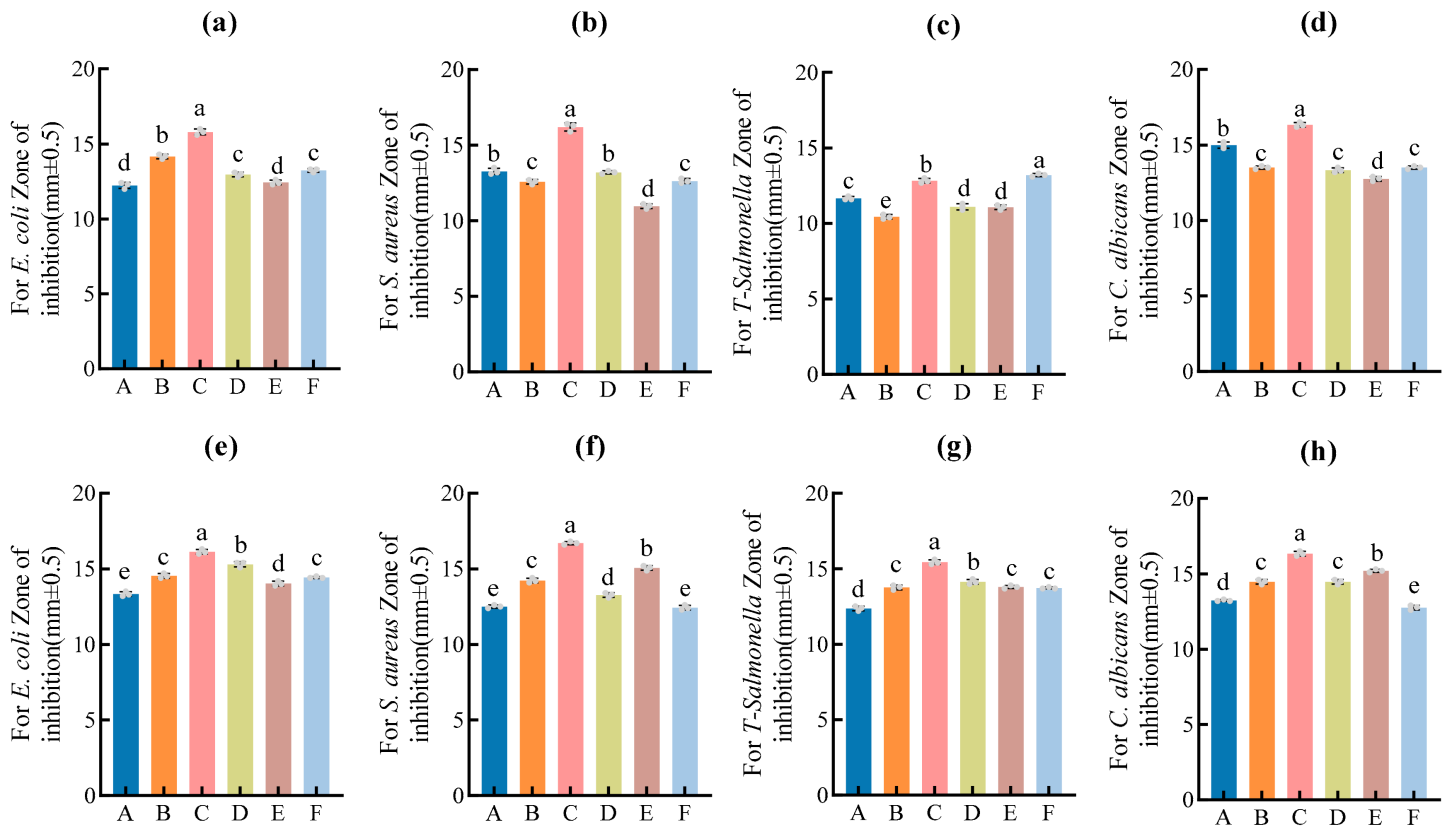
The inhibition effects of the drug on the extracts against four bacterial strains, (**a**–**d**) aqueous extracts; (**e**–**h**) hydroethanolic extracts; A. Herba *Centellae asiaticae* + Cortex *Betulae*, B. Flos *Calendulae* + Cortex *Betulae*, C. Folium *Aloe* + Cortex *Betulae*, D. Flos *Calendulae* + Folium *Aloe*, E. Flos *Calendulae* + Folium *Aloe*, and F. Flos *Calendulae* + Flos *Calendulae.* The letters a, b, c, d, e represent different levels of statistical significance.

The results of the aqueous extracts (Figure 2a–d) demonstrated that the Folium *Aloe* and Cortex *Betulae* combination exhibited the strongest and most broad-spectrum antibacterial activity across all four bacterial strains. The inhibitory zones against *E. coli* and *S. aureus* were particularly pronounced, highlighting the complementary antibacterial spectra of these two herbs: Folium *Aloe* contributed potent anti-Gram-negative and antifungal activity, whereas Cortex *Betulae* provided selective efficacy against Gram-positive bacteria [10,18]. The Flos *Calendulae* and Folium *Aloe* combination ranked second in overall potency, showing moderate activity against *S. aureus* and *C. albicans*. The remaining combinations exhibited relatively weaker inhibitory effects and no significant advantage over the optimal combination. The results of the alcohol extract (Figure 2e–h) confirmed the findings from the aqueous extract, demonstrating that the Folium *Aloe* and Cortex *Betulae* formulation once again exhibits superior antibacterial activity. Notably, the alcohol-extract combination formulation showed enhanced activity against *E. coli* and *S. aureus* compared to the aqueous extract formulation, consistent with the solvent-dependent efficacy observed in single-herb screening. This consistency across the dual-solvent system underscores the robust synergistic potential of the Folium *Aloe* and Cortex *Betulae* pairing. Quantitative analysis revealed that the minimum inhibition zone diameters for single-herb extracts were 11.1 mm (Folium *Aloe*) and 12.8 mm (Cortex *Betulae*), whereas the combined extract exhibited a maximum inhibition zone of 17 mm, representing a 21–36% enhancement over individual herbs. This synergistic effect validates the theoretical compatibility principle of “mutual support and complementarity” in traditional Chinese medicine.

Therefore, the Folium *Aloe* and Cortex *Betulae* combination was identified as the optimal formulation and proceeded to subsequent stages of extraction process optimization and comprehensive bioactivity evaluation.

### 2.3. Single-Factor Optimization of Folium Aloe and Cortex Betulae

After identifying Folium *Aloe* and Cortex *Betulae* as the optimal medicinal combination, systematic optimization of the extraction process parameters was required to maximize the antibacterial activity of the extract. Single-factor experiments formed the foundation of response surface design; by sequentially altering three key factors (solvent concentration, mixing ratio, and extraction time, with the antibacterial zone diameter as the evaluation metric), preliminary optimal ranges for each factor were determined, providing experimental basis for subsequent Box–Behnken response surface design (Supplementary Figure S3).

#### 2.3.1. The Effect of Hydroethanolic Concentration on the Antibacterial Activity of the Extract

**Figure 3:**
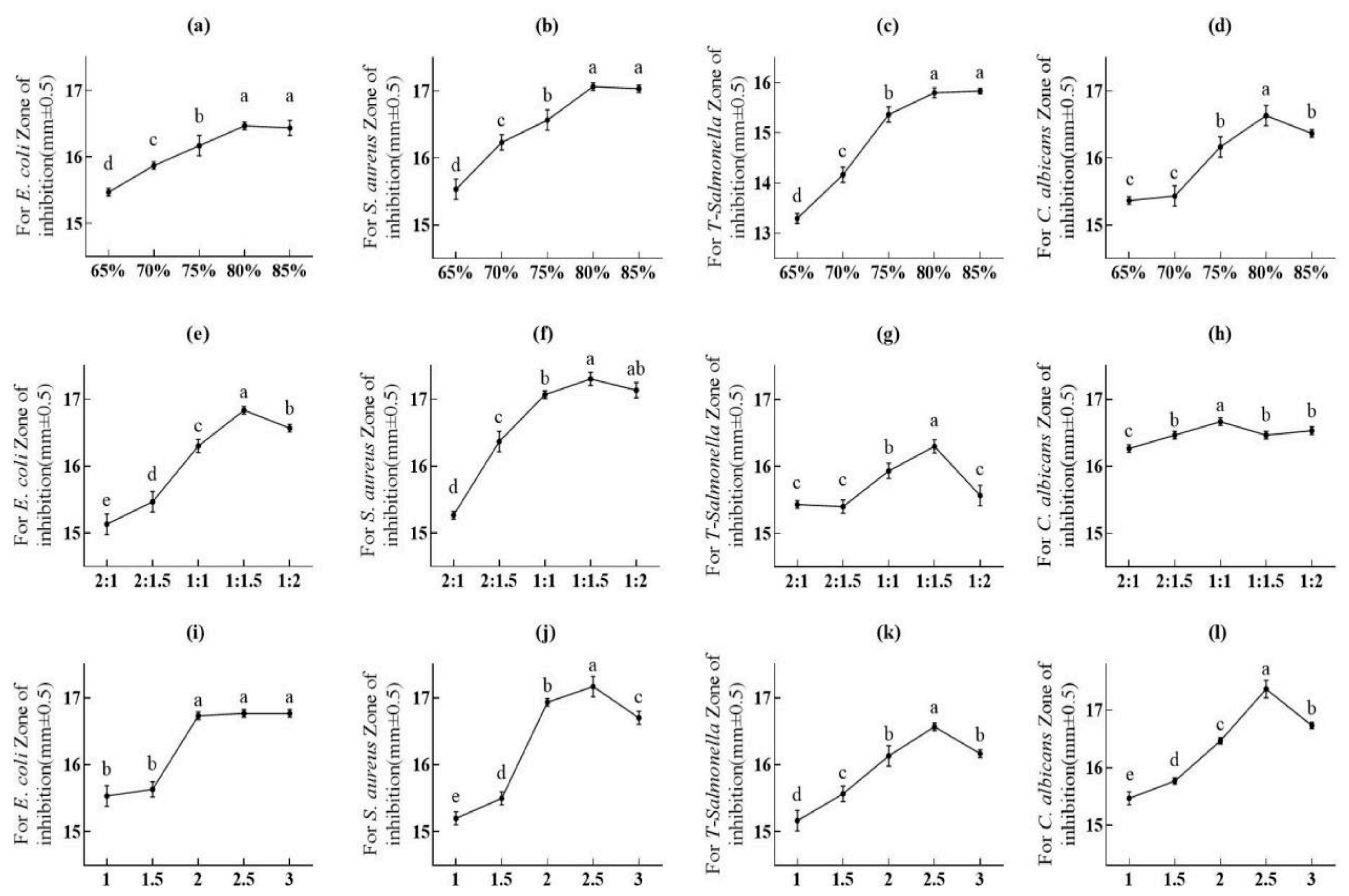
The antibacterial efficacy of Folium *Aloe* and Cortex *Betulae* extracts under varying conditions, (**a**–**d**): solvent concentration; (**e**–**h**) mass ratio; (**i**–**l**) extraction time. The letters a, b, c, d, e represent different levels of statistical significance.

Solvent concentration is one of the key factors influencing the extraction efficiency of plant active components, with different polar solvents exhibiting significant variations in their solubility for various chemical compounds. This study investigated the effects of five hydroethanolic concentration gradients (50%, 60%, 70%, 80%, and 90%) on the antibacterial activity of Folium *Aloe* and Cortex *Betulae* extract, as shown in Figure 3a–d. The experimental results demonstrated that the extract at an hydroethanolic concentration of 80% exhibited the largest inhibition zone diameter for all four test bacterial strains. This outcome is attributed to the increased solubility of lipophilic antibacterial compounds such as anthraquinones and flavonoids in a higher hydroethanolic concentration. Triterpenoids and phenolic compounds derived from Cortex *Betulae* exhibit optimal solubility in the polar environment of an 80% hydroethanolic aqueous solution. When the hydroethanolic concentration falls below 80%, the solvent becomes too polar to effectively dissolve lipophilic active ingredients. Conversely, elevated concentrations up to 90% reduce the extraction efficiency of certain polar auxiliary components such as polysaccharides and may induce excessive protein denaturation, thereby compromising the overall biological activity of the extract [21,22]. Consequently, 80% hydroethanolic was designated as the central level for subsequent response surface optimization.

#### 2.3.2. The Effect of the Formulation Ratio on the Antibacterial Efficacy of the Extract

The core of drug compatibility lies in the synergistic interaction between active components, whereas the formulation ratio directly determines the relative concentrations of each component in the extraction system, thereby influencing the efficacy of synergistic effects. This experiment established five mass ratio gradients of Folium *Aloe*: Cortex *Betulae* = 2:1, 1.5:1, 1:1, 1:1.5, and 1:2, with extraction performed under fixed conditions of 80% hydroethanolic concentration and extraction time. The results are shown in Figure 3e–h. The 1:1.5 ratio demonstrated optimal antibacterial efficacy under most test conditions. This advantage can be explained from a synergistic mechanism perspective: when the Folium *Aloe* proportion is too high, although Folium *Aloe* contains abundant polysaccharides, the relatively low levels of membrane-disrupting antimicrobial components such as birch alcohol in Cortex *Betulae* hinder the effective enhancement of the transmembrane transport of Folium *Aloe*’s active constituents. Conversely, an excessively high Cortex *Betulae* proportion increases membrane permeability but reduces the concentration of Folium *Aloe*’s anthraquinone compounds, thereby diminishing overall antibacterial potency [23,24]. The 1:1.5 ratio achieves a balanced interplay between the membrane-permeability-enhancing effect mediated by Folium *Aloe* polysaccharides and the direct membrane-dissolving effect of birch alcohol while maintaining optimal concentrations of both active components in the extract to maximize synergistic antibacterial efficacy.

#### 2.3.3. The Effect of the Extraction Time on the Antibacterial Efficacy of the Extract

The extraction time directly influences the dissolution rate and chemical stability of the active ingredients. An insufficient duration results in incomplete extraction, whereas prolonged exposure may lead to the degradation of heat-sensitive components. This study investigated five extraction time gradients (1, 1.5, 2, 2.5, and 3 h), with results shown in Figure 3i–l. During the initial extraction phase, as extraction time increased, active compounds such as *Aloe* anthraquinones and birch alcohol were progressively released, leading to sustained enhancement of the extract’s antibacterial activity. By 2.5 h, the primary antimicrobial components in the herbal material had been nearly fully extracted, achieving peak concentrations in the extract solution. If the extraction time is further extended to 3 h, certain thermosensitive components may undergo oxidative degradation or structural transformation due to prolonged heating, leading to reduced activity of the extract. Additionally, excessively prolonged extraction may increase the dissolution of inactive impurities in the herbal material, diluting the relative concentration of active constituents and thereby adversely affecting its antibacterial efficacy [25,26].

Based on the above single-factor experimental results, the optimal process parameters for Folium *Aloe* and Cortex *Betulae* extract were preliminarily determined as follows: a hydroethanolic concentration of 80%, a blending ratio of 1:1.5, and an extraction time of 2.5 h. However, the single-factor experiments only examined the independent effects of each factor and failed to reveal their interactions. Therefore, building upon single-factor optimization, a Box–Behnken response surface design was employed to conduct three-level optimization across all three factors, aiming to establish mathematical models linking each factor to response values, accurately predict optimal extraction conditions, and verify factor interaction effects.

### 2.4. Response Surface Optimization of Folium Aloe and Cortex Betulae Extract

Building upon a single-factor experiment, the Box–Behnken design (BBD) implemented in Design-Expert 13.0 software was employed to conduct a three-factor, three-level response surface optimization of the Folium *Aloe* and Cortex *Betulae* extraction process. This study systematically investigated the effects of three factors—mixing ratio, hydroethanolic concentration, and extraction time—as well as their interactions on the antibacterial activity of the extract, and established a mathematical model to predict optimal process parameters (Supplementary Figure S4)

#### 2.4.1. Experimental Design and Result Analysis

Using Design-Expert 13 software, a regression analysis was performed on the data in Table 1, yielding the following regression equation for *E. coli*: Y1 = 1.77 + 0.0083X1 − 0.0063X2 + 0.0021X3 − 0.0083X1X2 − 0.0167X1X3 + 0.0042X2X3 − 0.0637X1^2^ − 0.0262X2^2^ − 0.0513X3^2^.

*S. aureus*: Y2 = 1.88 − 0.0083 X1 − 0.0062 X2 + 0.0021 X3 + 0.0125 X1 X2 + 0.0125 X1 X3 + 0.0250 X2 X3 − 0.0942 X1^2^ − 0.0317 X2^2^ − 0.0400 X3^2^

*T-Salmonella*: Y3 = 1.75 + 0.0438 X1 + 0.0021 X2 + 0.0042 X3 − 0.0083 X1 X2 + 0.0125 X1 X3 − 0.0208 X2 X3 − 0.0642 X1^2^ − 0.0392 X2^2^ − 0.0267 X3^2^

*C. albicans*: Y4 = +1.89 + 0.0208 X1 + 0.0208 X2 + 0.0125 X3 + 0.0083 X1 X2 + 0.0083 X1 X3 − 0.0167 X2 X3 − 0.0675 X1^2^ − 0.0842 X2^2^ − 0.0425 X3^2^

#### 2.4.2. Experimental Design and Regression Model Establishment

All regression models achieved statistical significance (*p* < 0.01), indicating that the independent variables significantly influenced each response value; the residual terms were not significant (*p* > 0.05), suggesting that the model residuals stemmed solely from random errors and the model demonstrated good fit. The coefficient of determination (R^2^) values were 0.9265, 0.9783, 0.9394, and 0.9399, respectively, indicating a high correlation between model predictions and experimental measurements, thereby fully validating the model’s reliability. Among these, the *S. aureus* model exhibited the highest R^2^ value (0.9783), confirming its superior predictive accuracy for *S. aureus* inhibition rates.

#### 2.4.3. Intuitive Analysis of Response Surface Diagrams

**Figure 4:**
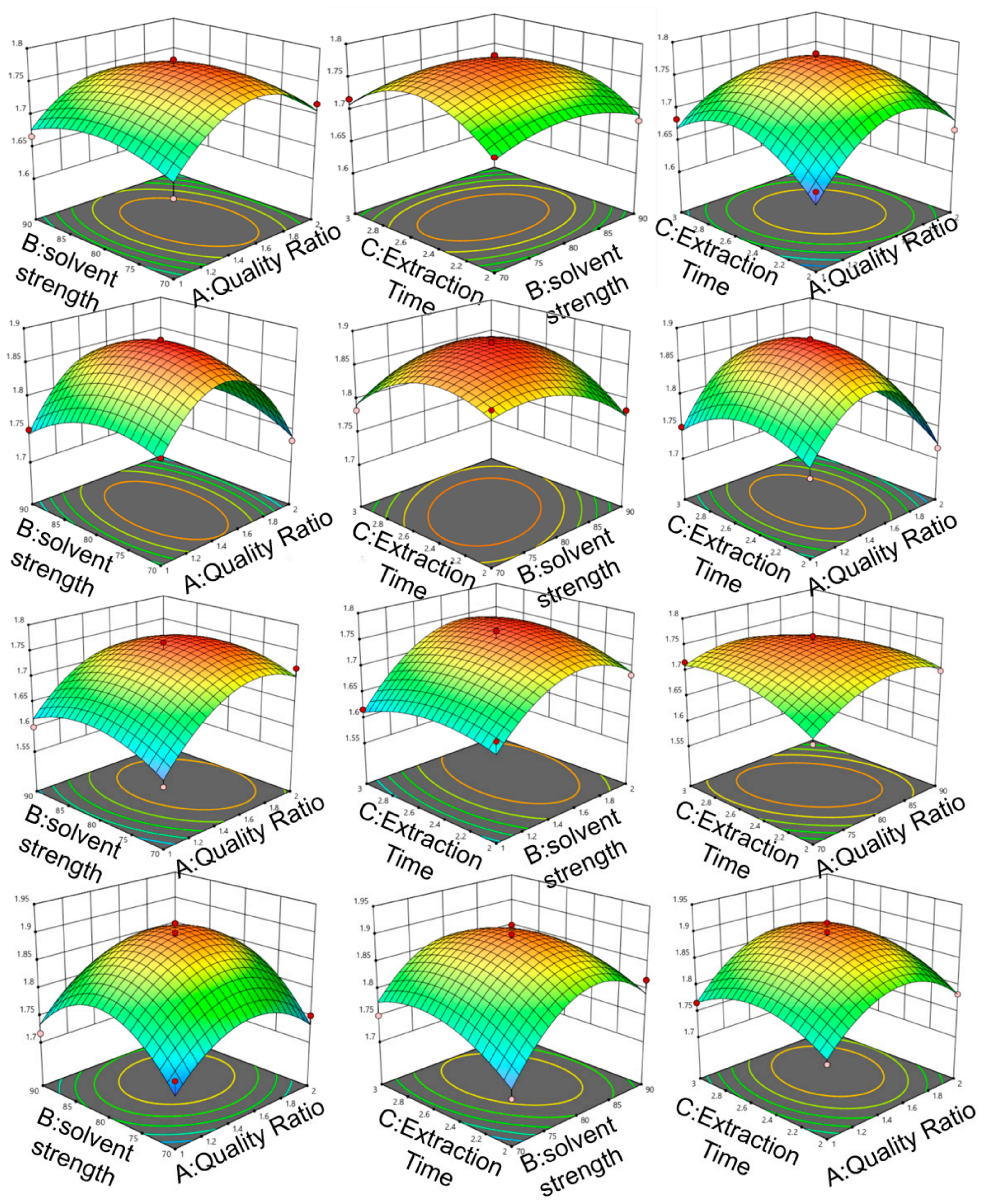
Diagram of the response surface model for various factors.

A three-dimensional response surface plot was constructed based on the regression equation (Figure 4) to visually analyze the effects of formulation ratios, hydroethanolic concentration, and extraction time on the inhibitory efficacy of the four bacterial strains, as well as their interactions (Supplementary Figure S5).

(1) The response surface plot of the interaction between the blending ratio and the hydroethanolic concentration demonstrates that this interaction significantly affects the inhibition rates for all bacterial strains. Under fixed extraction time conditions, as hydroethanolic concentration increased from 70% to 80%, all response values showed an upward trend; however, a further increase to 90% resulted in a decrease in inhibition rates, exhibiting a distinct parabolic pattern consistent with the single-factor experiment finding that 80% hydroethanolic was optimal. Regarding blending ratios, the region around 1:1.5 exhibited the highest response values, and deviations from this central point led to reduced inhibition rates, confirming the superiority of the 1:1.5 ratio identified in the single-factor experiment. On the contour plot, contours for the 1:1.5 ratio and the hydroethanolic concentration range of 79–80% were densely clustered and elliptical, indicating a significant interaction between these two factors.
(2) The interaction between the formulation ratio and the extraction time was equally significant. Around an extraction time of 2.5 h, all response parameters reached their peaks. An excessively short extraction time resulted in incomplete component dissolution, whereas a prolonged extraction time caused the degradation of heat-sensitive components. The optimal formulation ratio range was concentrated between 1.4 and 1.6, closely aligning with the single-factor analysis results. Notably, for *T-Salmonella* (Y_3_), the linear coefficient of the formulation ratio (0.0438) was significantly higher than that of other response parameters, indicating that this strain exhibited the highest sensitivity to variations in the formulation ratio, suggesting that the synergistic effect of Folium *Aloe* and Cortex *Betulae* on inhibiting *T-Salmonella* demonstrates distinct proportionality dependence.

(3) The interaction between hydroethanolic concentration and extraction time: The interaction effect between hydroethanolic concentration and extraction time was relatively weak but still reached a significant level. The contour plot shows that the combination of 80% hydroethanolic and 2.5 h of extraction time yielded the highest response value, with a narrow optimal range for both factors, indicating that precise control of the process parameters significantly influences extract activity.

Based on a comprehensive analysis of the standardized regression coefficients for all factors and the curvature of the response surface, the ranking of factors influencing the antibacterial activity of the extract is as follows: formulation ratio > hydroethanolic concentration > extraction time. The formulation ratio serves as the core variable determining drug compatibility, and its variation plays a decisive role in achieving synergistic effects. Hydroethanolic concentration ranks second, directly affecting the dissolution efficiency of active ingredients. The impact of extraction time is relatively minor but still requires precise control to prevent component degradation.

#### 2.4.4. Determination and Validation Tests of Optimal Process Parameters

The parameters of the optimal extraction process predicted by the regression model are as follows: a mixture ratio of 1.53398, a solvent concentration of 79.4609%, and an extraction time of 2.59714 h. The predicted inhibition rates for *E. coli*, *S. aureus*, *C. albicans*, and *T-Salmonella* were 177.39%, 187.92%, 175.60%, and 189.34%, respectively. For practical application convenience, the data were adjusted to a mixture ratio of 1.53, with a solvent concentration of 79.5% and an extraction time of 2.6 h. Three parallel validation experiments were conducted using the optimized process parameters, yielding predicted inhibition rates of 179.27% ± 0.12%, 189.92% ± 0.53%, 179.00% ± 0.34%, and 189.38% ± 0.10% for *E. coli*, *S. aureus*, *C. albicans*, and *T-Salmonella*, respectively. These results closely match the predicted values, demonstrating the reliability of the response surface model and its practical applicability.

### 2.5. UPLC-QTOF-MS Analysis

#### 2.5.1. Total Ion Current Chromatogram Analysis

**Figure 5:**
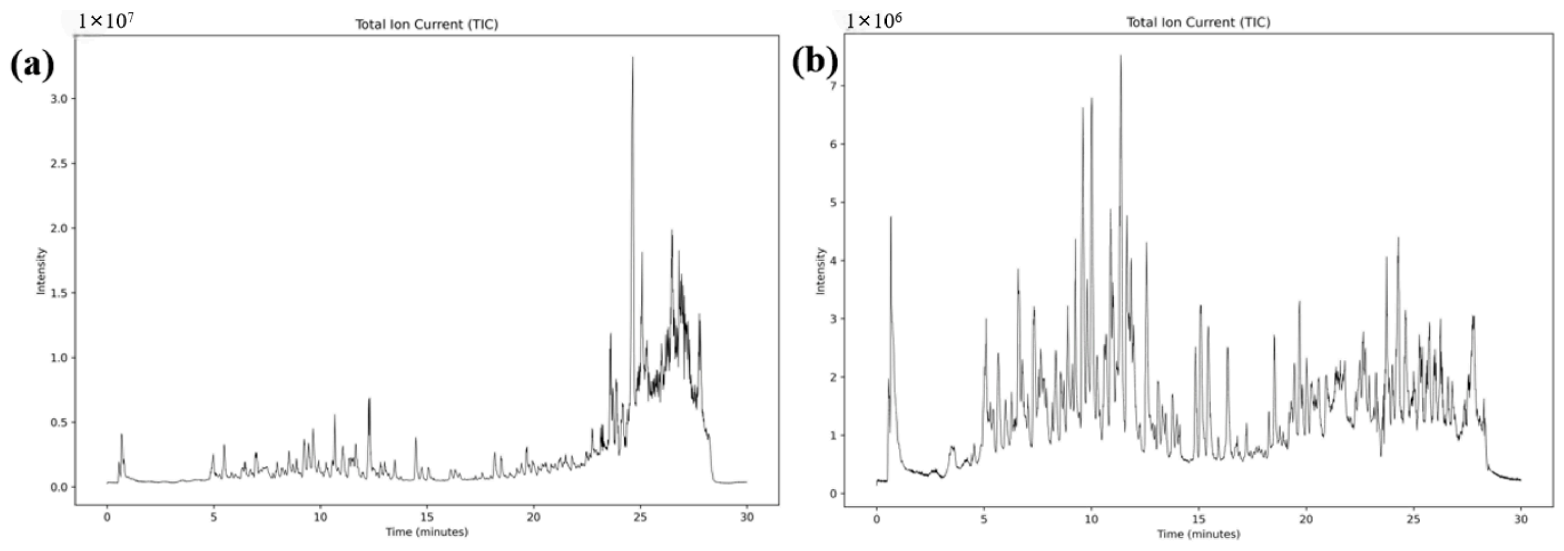
UPLC-QTOF-MS total ion chromatograms of the optimized Folium *Aloe* and Cortex *Betulae* extract. (**a**) Positive ion mode; (**b**) negative ion mode.

UPLC-QTOF-MS analysis was performed on the optimized Folium Folium *Aloe* and Cortex *Betulae* platyphyllae extract in both positive and negative ion modes. The positive ion mode total ion chromatogram (TIC, Figure 5a) demonstrated the effective separation of chemical constituents within 0–30 min, with major chromatographic peaks concentrated in two time windows: 5–15 min and 22–28 min. The most intense signal appeared at approximately 24 min, with a peak intensity of 3.0 × 10^7^. The negative ion mode TIC (Figure 5b) showed a more uniform distribution of compounds, with a prominent peak cluster at 10–12 min (peak intensity 7.5 × 10^6^) and additional signals at 0 min, 15–20 min, and 24–28 min. Good peak shapes and adequate resolution in both ion modes confirmed the suitability of this method for comprehensive chemical characterization of the extract [27,28,29].

#### 2.5.2. Compound Identification

Based on accurate molecular mass, retention time, characteristic fragment ions, and adduct ion information, combined with literature reports, eight major chemical constituents were identified from the extract, encompassing anthraquinones, triterpenoids, and chromones. Detailed mass spectrometric information is presented in Table 2.

Anthraquinones: In the negative ion mode, aloin was detected at 9.62 min as [M − H]^−^ *m*/*z* 417.1202 (ppm = 2.62), with characteristic fragment ions at *m*/*z* 297.0681 and 268.0675, corresponding to glycosidic bond cleavage and subsequent dehydration to form the aglycone fragment. In the positive ion mode, aloin was detected at 9.65 min as [M + H]^+^ *m*/*z* 419.1337 (ppm = 0.09), producing fragments at *m*/*z* 257.0773, 239.0692, and 211.0747, attributed to protonated aloin aglycone and its consecutive dehydration products. Aloe-emodin and emodin share the same molecular formula (C_15_H_10_O_5_) and were both detected as [M − H]^−^ at *m*/*z* 269.0455 and 269.0479 in the negative ion mode. Aloe-emodin (RT 18.45 min, ppm = 0.18) exhibited a fragment series at *m*/*z* 269.0354, 241.0435, 227.0315, and 225.0459, corresponding to [M − H]^−^ loss of CO, CO_2_, and consecutive dehydration. Emodin (RT 12.13 min) produced characteristic fragments at *m*/*z* 269.0402 and 227.0338. These anthraquinone identifications are consistent with the established phytochemical profile of Folium Aloe vera [30,31].

Chromones: Aloeresin A was detected in both ion modes. In the positive mode, it was detected at 8.24 min as [M + H]^+^ *m*/*z* 541.1725 (ppm = 3.80), with fragments at *m*/*z* 395.1304 and 275.0923, indicating ester bond cleavage between the chromone nucleus and cinnamoyl group, and *m*/*z* 147.0446 corresponding to the cinnamic acid moiety. In the negative mode, it was detected at 8.60 min as [M − H]^−^ *m*/*z* 539.1522 (ppm = 6.84), producing fragments at *m*/*z* 375.0984 and 163.0380, further confirming the structural assignment. This chromone glycoside is a characteristic constituent of Aloe species [29,32].

Triterpenoids: The Cortex *Betulae*-derived triterpenoids betulinic acid and betulin were detected in the positive ion mode at 21.16 min and 22.52 min, respectively. Betulinic acid yielded [M + H]^+^ *m*/*z* 457.3676 with a mass error of only 0.05 ppm; betulin yielded [M + H]^+^ *m*/*z* 443.3881 (ppm = 0.58), with its MS/MS fragment at *m*/*z* 191.1786, representing a characteristic triterpenoid cleavage product. Oleanolic acid was detected in negative ion mode at 26.71 min as [M − H]^−^ *m*/*z* 455.3524 (ppm = 1.47), with rich fragment ions including *m*/*z* 455.3427 (precursor), 437.3237 ([M − H−H_2_O]^−^), 411.3392 ([M − H−CO_2_]^−^), 409.3270, and 395.3209, corresponding to dehydration and decarboxylation pathways consistent with the established fragmentation pattern of oleanolic acid. These triterpenoid identifications confirm the presence of Cortex Betulae platyphyllae constituents in the optimized extract [31,33].

In summary, the UPLC-QTOF-MS method established in this study effectively separated and identified the major bioactive constituents from Folium *Aloe* and Cortex *Betulae*. The identified compounds span anthraquinones, chromones, and triterpenoids, with mass accuracy better than 10 ppm for all constituents, meeting the qualitative analysis requirements of high-resolution mass spectrometry. These phytochemical data provide the necessary chemical foundation for correlating biological activity with specific constituents, addressing the standardization concerns raised in the methodological framework.

### 2.6. Biocompatibility Evaluation of the Optimized Extract

**Figure 6:**
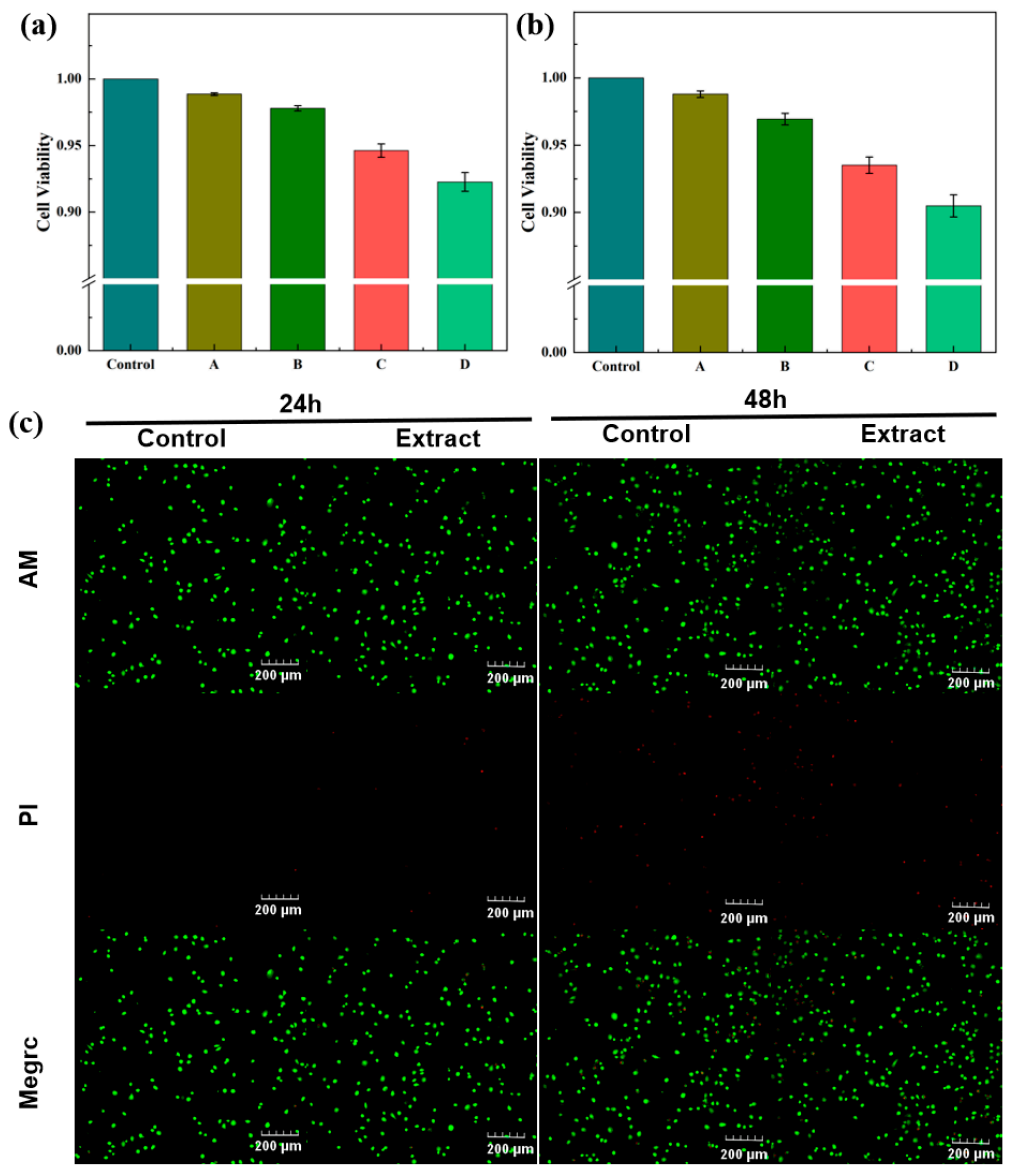
Biocompatibility: (**a**,**b**) Cell viability of L929 cells treated with varying concentrations of Folium *Aloe* and Cortex *Betulae* extracts at 24 and 48 h; (**c**) Comparative staining image of live versus dead cells. A–D represent Folium *Aloe* and Cortex *Betulae* extracts at concentrations of 100, 200, 500, and 1000 μg/mL, respectively.

Figure 6a presents the quantitative assessment of L929 fibroblast viability following 24 h exposure to serial concentrations of the optimized Folium *Aloe* and Cortex *Betulae* extract. Cell viability remained remarkably high across all tested concentrations, with the control group normalized to 1.000. At 100 μg/mL, the viability was 0.9886 ± 0.0010, statistically indistinguishable from the control. At 200 μg/mL, the viability was 0.9778 ± 0.0020, demonstrating minimal cytotoxic effect. A modest concentration-dependent decline was observed at higher concentrations: 0.9463 ± 0.0050 at 500 μg/mL and 0.9227 ± 0.0071 at 1000 μg/mL. All values exceeded the ISO 10993-5 [34] biocompatibility threshold of 75%, with even the highest concentration maintaining >92% viability.

Prolonged exposure of 48 h (Figure 6b) revealed a similar declining trend with marginally reduced viability values. At 100 μg/mL and 200 μg/mL, cell viability remained 0.9879 ± 0.0025 and 0.9693 ± 0.0043, respectively, affirming the absence of subacute cytotoxicity. At 500 μg/mL and 1000 μg/mL, the viability was 0.9352 ± 0.0061 and 0.9049 ± 0.0082, respectively, representing a statistically significant but biologically inconsequential reduction compared to the 24 h timepoint. The live/dead fluorescence staining (Figure 6c) corroborated these quantitative findings, displaying predominant green fluorescence (calcein-AM, viable cells) with a negligible red signal (PI, dead cells) across all concentrations and both timepoints, confirming intact cellular membrane integrity and metabolic activity.

### 2.7. Evaluation of the Antibacterial Activity of the Extract

#### 2.7.1. Analysis of Antibacterial Performance

The optimal process extract was compared with the drug extract obtained before single-factor optimization (mixing ratio 1:1, 75% hydroethanolic, 3 h). After optimization, the inhibition rates of the four strains increased by 3.93% (*E. coli*), 4.79% (*S. aureus*), 8.64% (*T-Salmonella*), and 6.33% (*C. albicans*), respectively. Among them, *T-Salmonella* exhibited the highest increase rate (8.64%), likely due to its high sensitivity to formulation ratios. Response surface optimization precisely tuned the ratio to 1.53:1, significantly enhancing the synergistic inhibitory effect of *Aloe* polysaccharides and birch alcohol against this strain (Table 3).

**Figure 7:**
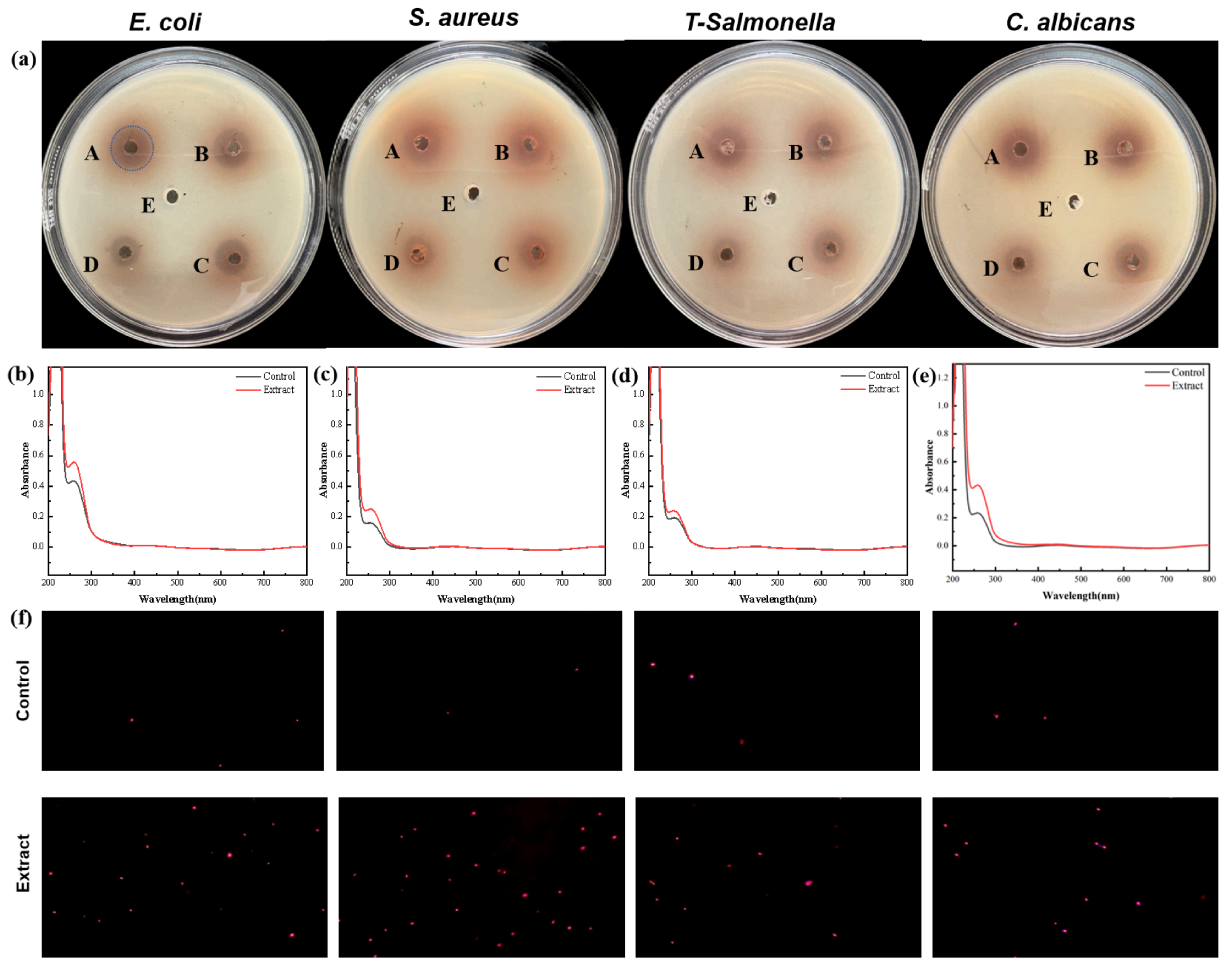
Bacteriostatic properties of Folium *Aloe* and Cortex *Betulae* extract and their mechanisms. (**a**) Actual image of agar diffusion (ABCD represent extracts with concentrations of 1, 0.8, 0.6, and 0.4 mg/mL, respectively; E denotes the blank control group). (**b**–**e**) UV-Vis absorption spectra of the bacterial supernatants at 260 nm (nucleic acids) and 280 nm (proteins); (**f**) propidium iodide (PI) staining of the bacteria showing membrane integrity compromise.

The optimized extract exhibited a bacterial inhibition zone diameter of 17.5–19.0 mm, representing a significant improvement over the initial process (approximately 16.5–17.5 mm), with a low standard deviation in inhibition rates against four bacterial strains (0.10–0.53%), demonstrating excellent reproducibility and stability of the optimized process. These results confirm that the combination of single-factor experiments and the Box–Behnken response surface design effectively enhance the antibacterial activity of the Folium *Aloe* and Cortex *Betulae* extract (Figure 7a).

Folium *Aloe* exhibits a MIC of 50 mg/mL against both *E. coli* and *C. albicans*, 31.25 mg/mL against *S. aureus*, and the lowest MIC of 25 mg/mL against *T-Salmonella*, indicating its relative superiority against Gram-negative bacteria and fungi. The MIC of Cortex *Betulae* against *E. coli* and *S. aureus* was 31.25 mg/mL, whereas it reached as high as 100 mg/mL against *T-Salmonella* and 62.5 mg/mL against *C. albicans*. These results indicate that Cortex *Betulae* exhibits significant efficacy against Gram-positive bacteria and most Gram-negative bacteria but demonstrates weaker inhibitory effects against *T-Salmonella* and fungi. This difference in the antibacterial spectrum provides the experimental basis for the complementary synergy of the two drugs when used together.

The optimized Folium *Aloe* and Cortex *Betulae* combination extract exhibited lower MIC values for all four bacterial strains compared to individual components, demonstrating a clear synergistic effect. Specifically, the MIC against *S. aureus* decreased from 31.25 mg/mL with the single component to 15.625 mg/mL, representing a reduction of 50%. The MIC of *C. albicans* decreased significantly from 50 mg/mL in Folium *Aloe* and 62.5 mg/mL in Cortex *Betulae* to 15.625 mg/mL, representing reductions of 68.75% and 75%, respectively. This significant synergistic effect is attributed to the fact that aloe polysaccharides enhance the membrane permeability of birch alcohol, facilitating its entry into bacterial cells for targeted action, whereas birch alcohol stabilizes the anthraquinone components in *Aloe* and delays their oxidative degradation.

The MBC assay results demonstrated that the ratio of MBC to MIC (MBC/MIC) for the optimized combined extract was ≤3, meeting the criteria for a bactericidal agent, indicating that the extract not only exhibits antibacterial activity but also possesses reliable bactericidal efficacy (Supplementary Figure S6) [35,36]. This further confirms that the optimized Folium *Aloe* and Cortex *Betulae* extract exhibits bactericidal activity against all four pathogenic bacteria, rather than merely serving as an antibacterial agent that inhibits bacterial growth.

#### 2.7.2. Preliminary Exploration of Bacteriostatic Mechanisms

To preliminarily investigate the damage mechanism of the optimized extract on bacterial cells, ultraviolet-visible spectrophotometry was employed to detect nucleic acid and protein leakage in bacterial suspensions, supplemented by propidium iodide (PI) staining assays to directly visualize bacterial membrane integrity (Figure 7b–f).

The UV-Vis spectrophotometry results demonstrated that the optimized extract significantly disrupted the integrity of *S. aureus* and *E. coli* cell membranes at MIC concentrations, leading to substantial leakage of intracellular nucleic acids and proteins. The marked increase in absorbance at 260 nm indicated the release of DNA and RNA, whereas the rise at 280 nm reflected the exudation of proteinaceous substances. These results indicate membrane integrity disruption under our experimental conditions, consistent with established protocols for detecting cytoplasmic leakage.

PI staining provided direct visual evidence of membrane compromise. PI is a membrane-impermeant nucleic acid dye that selectively stains cells with compromised membrane integrity. Fluorescence microscopy images revealed a substantial increase in PI-positive (red fluorescent) bacterial cells in the extract-treated groups compared to blank controls for both *E. coli* and *S. aureus*, confirming that the optimized extract significantly compromises bacterial membrane integrity, leading to increased cell permeability and subsequent cell death.

The combination of direct membrane integrity visualization (PI staining) and quantitative macromolecular leakage analysis (UV spectroscopy) provides converging evidence supporting membrane disruption as a key antibacterial mechanism. The lipophilic pentacyclic triterpenoids (e.g., betulin) from Cortex *Betulae* can insert into the bacterial cell membrane’s phospholipid bilayer, alter membrane fluidity, induce membrane perforation, and cause intracellular substance leakage. Anthraquinone compounds in Folium *Aloe* may synergistically enhance membrane damage by chelating metal ions and inhibiting membrane-associated enzyme activity. These mechanistic interpretations are supported by our experimental evidence of membrane disruption and are consistent with the established antibacterial mechanisms of these compound classes [37,38].

### 2.8. Evaluation of the Optimal Drug’s Antioxidant Activity on the Extract

Impaired healing of chronic wounds is closely associated with the local oxidative stress microenvironment. The excessive accumulation of reactive oxygen species (ROS) can induce lipid peroxidation in cell membranes, protein denaturation, and DNA damage, thereby exacerbating inflammatory responses and impairing tissue regeneration [39,40]. Therefore, evaluating the antioxidant activity of optimized Folium *Aloe* and Cortex *Betulae* extracts is crucial for elucidating their wound-healing mechanisms and expanding their clinical applications. This study systematically assessed the antioxidant capacity of both single-herb extracts and optimized combined extracts using DPPH and ABTS free radical scavenging assays.

**Figure 8:**
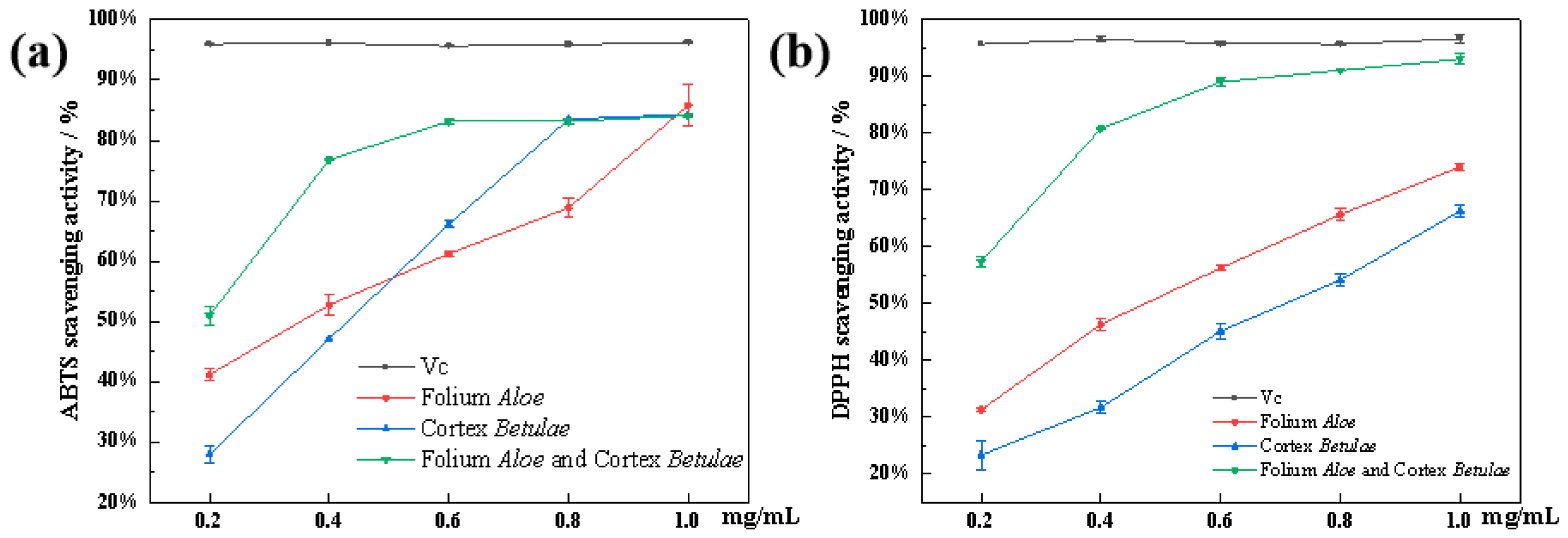
Antioxidant Performance Analysis. (**a**) ABTS, (**b**) DPPH.

The experimental results are shown in Figure 8a, where the DPPH scavenging rates of Folium *Aloe* and Cortex *Betulae* and their combined extract all exhibited a significant concentration-dependent increase. At a concentration of 1.0 mg/mL, the DPPH scavenging rates reached approximately 70% for Folium *Aloe*, 65% for Cortex *Betulae*, and 90% for the combined extract. Notably, the scavenging rate of the combined extract was significantly higher than that of each individual extract at all tested concentrations, demonstrating a pronounced synergistic antioxidant effect. In the low-concentration range (0.2–0.6 mg/mL), the synergistic effect of the combined extract was particularly pronounced, with its scavenging rate exceeding the theoretical sum of the individual components by 15–25%, suggesting the formation of new antioxidant active sites or enhanced electron transfer efficiency of the constituent components.

The experimental results are shown in Figure 8b, where the ABTS scavenging rate of the Folium *Aloe* and Cortex *Betulae* combination extract was comparable to that of the Vc (ascorbic acid) positive control. Within the concentration range of 0.2–1.0 mg/mL, the ABTS scavenging rate of the combination extract steadily increased from 45% to levels equivalent to those of Vc, whereas the scavenging rates of individual herb extracts remained consistently lower. These findings further confirm the synergistic antioxidant effect of the combination and demonstrate its high efficacy in scavenging both water-soluble and lipid-soluble free radicals (Figure 8).

In this study, the Folium *Aloe* and Cortex *Betulae* combination extract demonstrated significant synergistic effects in both antioxidant and antibacterial dimensions. This dual synergistic effect phenomenon stems from the shared chemical basis of these two bioactive compounds, polyphenols and flavonoids. The anthraquinone compounds in Folium *Aloe* also possess both antioxidant and antibacterial functions, and their redox activity enables them to address both oxidative stress and microbial infections.

## 3. Materials and Methods

### 3.1. Materials

The following herbal raw materials were used: Cortex *Bet*ulae (collected from Tahe Town, Tahe County, Greater Khingan Range, Heilongjiang Province), and Herba *Centellae asiaticae*, Flos *Calendulae*, Folium *Aloe* and Rhizoma *Curcumae longae*, which were all sourced from the Chengdu Hehuachi Herbal Raw Materials Market (Chengdu, China). All these herbal raw materials were identified by Professor Zhang Chenlu from the School of Biological Science and Engineering at Shaanxi University of Technology before use.

*Aloe vera* clarification: Although Gel *Aloe* (leaf pulp extract) is predominantly used in dermatological and cosmetic preparations for its moisturizing polysaccharides, and Succus *Aloe* (fresh leaf juice) is employed in oral supplements, Folium *Aloe* (dried leaf) provides a broader spectrum of bioactive constituents, including anthraquinones, flavonoids, and polysaccharides, making it suitable for comprehensive antibacterial screening [32,41].

Anhydrous ethanol (AR, Lianlonghua Pharmaceutical Chemical Co., Ltd., Tianjin, China); sodium chloride (AR, Tianjin Tianli Chemical Reagents Co., Ltd., Tianjin, China); tryptone, agar, yeast extract powder (BR, Tianjin Tianli Chemical Reagents Co., Ltd.) were used. Both acetonitrile and methanol are chromatographically pure and were purchased from Merck (Darmstadt, Germany). Formic acid (chromatographically pure) was obtained from CNW (Shanghai, China). *E. coli*, *S. aureus*, *C. albicans*, and *T-Salmonella* (Shaanxi Edible Fungi Research Institute, Xi’an, China) were also used.

### 3.2. Selection of Herbal Raw Materials

#### 3.2.1. Extraction of Active Components from Herbal Raw Materials

All herbal raw materials were subjected to standardized pre-treatment: low-temperature drying at 40–45 °C in a DHG-9070A electric thermostatic drying oven (Shanghai Yiheng Scientific Instruments Co., Ltd., Shanghai, China) to moisture content <10%, followed by grinding in a FW-100 high-speed universal grinder (Tianjin Taisite Instrument Co., Ltd., Tianjin, China) and sieving through a 50-mesh standard sieve (aperture 300 μm). The processed powders were stored in sealed desiccators at room temperature and used within 6 months of drying to minimize the degradation of thermosensitive constituents.

Fifty grams of each powder was weighed and added to 500 mL of ultrapure water and 75% hydroethanolic, respectively, soaked overnight, then subjected to slow reflux extraction for 3 h. The extract was initially filtered through gauze, followed by centrifugation using a TGL-16M high-speed refrigerated centrifuge (Shanghai Lu Xiangyi Centrifuge Instrument Co., Ltd., Shanghai, China) at 10,000 rpm (≈12,000× *g*) for 10 min at 4 °C. The filtrate was concentrated to 50 mL using a RE-52AA rotary evaporator (Shanghai Yarong Biochemical Instrument Factory, Shanghai, China) at 40 °C under reduced pressure (0.08–0.09 MPa) with a SHZ-D(III) circulating water vacuum pump (Gongyi Yuhua Instrument Co., Ltd., Zhengzhou, China). For the hydroethanolic extracts, hydroethanolic was completely removed during concentration. The residue was reconstituted in 50 mL of fresh 75% hydroethanolic to ensure a standardized final solvent concentration. All extracts were standardized to a crude drug equivalent concentration of 1 g/mL (DER 1:1, *w*/*v*).

#### 3.2.2. Antibacterial Test

For the bacterial culture activation, 4 g of trypsin, 2.5 g of yeast extract powder, 5 g of sodium chloride, and 15 g of agar were dissolved in 500 mL of deionized water, then the mixture was sterilized in an autoclave at 121 °C for 30 min. Under sterile conditions, the sterilized culture medium hot was transferred into sterilized petri dishes and allowed to cool and solidify. This was then stored for later use. Using an inoculation loop, the bacterial suspension was streaked onto LB solid medium, followed by incubation at 37 °C for 12 h. Upon completion, individual colonies from the culture were selected, transferred to test tubes containing 5 mL of liquid medium, and incubated under constant stirring at 37 °C for another 12 h before storage in a refrigerator.

Bacterial culture: *E. coli*, *S. aureus*, *C. albicans*, and *T-Salmonella* were cultured in an incubator at 37 °C. The bacterial suspensions were adjusted to a concentration of 10^7^ CFU/mL for subsequent antimicrobial assays [42].

Agar well diffusion method: 50 μL of aqueous and hydroethanolic extracts from the herbal raw materials, respectively, was added into agar wells on plates inoculated with test bacteria. The plates were inverted and incubated in an incubator at 37 °C for 12 h, then the diameter of the inhibition zones was measured and recorded to evaluate the inhibitory effects of the five extracts against four bacterial strains, thereby identifying the herbs with significant antibacterial activity.

### 3.3. Screening of Drug Combinations

Based on the experimental results in Section 3.2, the screened herbs with relatively significant antibacterial activity were mixed using the grid method at a 1:1 mass ratio. After extraction, the inhibitory activities of both aqueous and hydroethanolic extracts against *E. coli*, *S. aureus*, *C. albicans*, and *T-Salmonella* were measured separately by agar well diffusion assay. A significance analysis was conducted to identify the drug combination pairs exhibiting statistically significant antibacterial efficacy.

### 3.4. Optimization of Extraction Conditions

#### 3.4.1. Single-Factor Optimization

Single-factor optimization experiments were conducted on the selected optimal drug combinations by altering the solvent concentration, extraction time, and formulation ratios of the herbal raw materials, with antibacterial efficacy as the evaluation criterion to determine the optimal extraction conditions. The single-factor variable table is shown in Table 4, with all other parameters kept constant.

#### 3.4.2. Response Surface Optimization

The experimental conditions were determined using the Box–Behnken design optimization method in Design-Expert 13.0 software. Based on the results of the aforementioned single-factor experiments, the inhibition rates of *S. aureus*, *E. coli*, *T-Salmonella* and *C. albicans* were employed as dependent variables. Three variables—mixing ratio (A), solvent concentration (B), and extraction time (C)—were selected as independent variables for the BBD, and a three-factor, three-level response surface optimization experiment was designed, as shown in Table 5.

### 3.5. UPLC-QTOF-MS

#### 3.5.1. Sample Preparation

The optimized Folium *Aloe* and Cortex *Betulae* extract was subjected to UPLC-QTOF-MS analysis for phytochemical characterization. An aliquot (100 μL) was transferred to a 1.5 mL EP tube, vortexed for 30 s, and mixed with 100 μL of 95% methanol. After centrifugation at 17,000× *g* for 10 min at 20 °C, the supernatant was transferred to a sample vial for UPLC-QTOF-MS analysis.

#### 3.5.2. UPLC-QTOF-MS Conditions

Chromatographic conditions: An ACQUITY UPLC HSS T3 column (Waters, Milford, MA, USA, 1.8 μm, 2.1 mm × 100 mm, Waters) was employed. The column temperature was maintained at 40 °C, the injection volume was 5 μL, and the flow rate was 400 μL/min. Mobile phase A consisted of 0.1% formic acid in water, and mobile phase B consisted of 0.1% formic acid in acetonitrile. The gradient elution program was as follows: 0.00–1.50 min, 5% B; 1.50–2.50 min, 5–10% B; 2.50–14.00 min, 10–40% B; 14.00–24.00 min, 40–95% B; 24.00–27.00 min, 95% B; 27.00–27.10 min, 95–5% B; 27.10–30.00 min, 5% B.

Mass spectrometric conditions: An AB 5600 Triple TOF mass spectrometer (AB SCIEX, Marlborough, MA, USA), controlled by Analyst TF 1.7 software, was operated in the information-dependent acquisition (IDA) mode for simultaneous MS and MS/MS data collection. In each acquisition cycle, the most intense precursor ions with an intensity >100 were automatically selected for MS/MS fragmentation. The MS scan range was *m*/*z* 50–1200, the collision energy was 30 eV, and 10 MS/MS spectra were acquired per cycle. ESI source parameters: nebulizer gas (GS1) 60 psi, auxiliary gas 60 psi, curtain gas 35 psi, source temperature 550 °C; spray voltage +5500 V (positive mode) or −4500 V (negative mode).

### 3.6. Biological Safety Testing

Cytotoxicity Assay: L929 mouse fibroblasts were cultured in Dulbecco’s Modified Eagle Medium (DMEM) supplemented with 10% fetal bovine serum (FBS) and 1% penicillin-streptomycin, and were maintained in a humid incubator at 37 °C with 5% CO2. When the cells reached 80% confluence, they were digested with 0.25% trypsin-EDTA, then subcultured and seeded at a density of 1 × 10^4^ cells per well in a 96-well plate, and incubated for 24 h to ensure complete cell adhesion [43]. The medium was then aspirated and replaced with fresh DMEM supplemented with extract at gradient dilutions, with final concentrations of 100, 200, 500 and 1000 μg/mL, respectively. Incubation was continued for 24 h and 48 h separately. After incubation, 10 μL of CCK-8 reagent was added to each well and incubated for 2 h under the same conditions. Subsequently, the absorbance at 450 nm was measured in each well using an enzyme-labeled instrument (Shandong Lainde Intelligent Technology Co., Ltd., Liaocheng, China) [44]. The cell viability was calculated using the blank control group cells treated with medium only without exposure to the samples as the reference.

(1) $$\mathrm{Cell}\;\mathrm{Viability}=\frac{\mathrm{Sample}\;\mathrm{group}\;\mathrm{absorbance}-\text{CCK-8}\;\mathrm{blank}}{\mathrm{Control}\;\mathrm{group}\;\mathrm{absorbance}-\text{CCK-8}\;\mathrm{blank}}\;\times \;100\%$$

Live/Dead Cell Staining: L929 mouse fibroblasts were seeded at a density of 5 × 10^5^ cells per well in a 6-well plate and incubated overnight to ensure complete adhesion. The cells were then transferred to fresh DMEM medium containing extract and co-incubated for 24 and 48 h, respectively. After incubation, the medium was aspirated, and the cells were gently rinsed twice with pre-cooled PBS to remove non-adherent cells and residual samples. Subsequently, the cells were stained for 15 min under light-shielded conditions at 37 °C using the Calcein AM/PI live/dead cell fluorescence staining kit (Servicebio, Wuhan, China). Finally, live cells (green fluorescence) and dead cells (red fluorescence) were observed and imaged using confocal laser scanning microscopy (CLSM, Olympus Corporation (Evident), Tokyo, Japan) [44]. This experiment was designed to further validate the cytotoxic characteristics of the samples.

### 3.7. Determination of the Optimal Drug Combination’s Antibacterial Efficacy on Extracts

#### 3.7.1. Agar Diffusion Assay

Using *E. coli*, *S. aureus*, *C. albicans*, and *T-Salmonella* as test strains, the antibacterial efficacy of the extracts against these bacteria was qualitatively evaluated by the agar diffusion method. The required materials were sterilized at 121 °C for 30 min, then the extracts were diluted with 75% hydroethanolic to concentrations of 1, 0.8, 0.6, and 0.4 mg/mL for subsequent use, with 75% hydroethanolic serving as the control to assess the antibacterial performance of the extracts. The overnight-activated test bacteria were diluted with deionized water to a concentration of 5 × 10^7^ CFU/mL, and 400 μL was evenly spread onto solid culture medium. A 6-mm agar well was created on the medium surface and 50 μL of samples at different concentrations along with control samples were added to each well. Three parallel experiments were conducted, and the plates were incubated at 37 °C for 12 h before the results were observed and photographed using a digital camera.

#### 3.7.2. MIC and MBC

The microdilution method in 96-well plates was used to determine the MIC of the samples. A total of 100 μL of sterile LB liquid medium was added to wells 2–12 of the 96-well plate, and 200 μL of the sample solution was added to well 1; two-fold serial dilution was performed from well 1 to well 12, and 100 μL of the solution in well 12 was discarded after mixing. A total of 100 μL of bacterial suspension (1 × 10^7^ CFU/mL) was added to each well, with positive control wells (bacterial suspension + medium) and blank control wells (medium only) set up. The plates were incubated at 37 °C for 24 h, and the lowest sample concentration with no visible bacterial growth was defined as the MIC. For MBC determination, the sample solutions at the MIC and adjacent concentrations were pipetted onto LB solid medium plates, and 5 μL of each solution was added dropwise to three replicate points on the plate. Negative and positive controls were set up simultaneously, and the plates were incubated at 37 °C overnight. The lowest sample concentration with no bacterial colony growth on the plate was defined as the MBC [36,38].

#### 3.7.3. Investigation of Antibacterial Mechanisms

The characteristic absorption peaks of the solutes could be detected at 260 nm using a UV-Vis spectrophotometer (Shanghai Yuanxi Instrument Co., Ltd., Shanghai, China), enabling further investigation into the antibacterial mechanism of the extract. First, the four test bacterial strains that had been activated overnight were diluted to 1.5 × 10^8^ CFU/mL for subsequent use. A total of 3 mL of bacterial suspension was transferred to a centrifuge tube as the experimental group. The extract was added to reach a final concentration of 0.4 mg/mL. The prepared mixture was incubated under ambient conditions at 25 °C for 24 h and then centrifuged at 15,000 rpm for 10 min. Finally, the supernatant was filtered using a 0.22 µm filter to remove residual viable bacteria. Additionally, 3 mL of bacterial suspension was taken as the control group, which received identical treatment to the experimental group except for the absence of extract. Finally, the ultraviolet spectral absorption values of the filtrate were measured at 260 nm using a UV-Vis spectrophotometer. Data were recorded and analyzed for all three groups to assess bacterial cytoplasmic leakage.

The bacterial suspension (1 × 10^8^ CFU/mL) was co-cultured with extract at 37 °C for 12 h, then centrifuged at 5000 rpm for 5 min to collect the bacterial precipitate. The bacteria were stained with PI staining solution for 15 min, centrifuged to remove the supernatant, and washed three times with PBS. A 5 μL drop of the bacterial suspension was placed on a glass slide, and the red fluorescence was observed and imaged under a fluorescence microscope. The blank control group was set up for comparison.

### 3.8. Antioxidant Activity Test of the Optimal Drug-Drug Pair on Extracts

#### 3.8.1. DPPH Free Radical Scavenging Assay

A total of 5 mg of DPPH was weighed, dissolved in anhydrous hydroethanolic by ultrasonication under light protection, and diluted to 100 mL to prepare the DPPH solution for immediate use. In a 2 mL centrifuge tube, the herbal extract sample and DPPH solution was added at a ratio of 1:3 (*v*/*v*). The mixture was left to react under light protection at room temperature for 30 min and was then centrifuged at 10,000 rpm for 3 min [39]. The clear supernatant was transferred to a 96-well plate, with three replicates per sample. The absorbance was measured at 517 nm and the value was calculated as As.

Using the sample solvent as a reference, the absorbance of the herbal extract mixed with the sample solvent at a ratio of 1:3 (*v*/*v*) was measured and recorded as Ac. Simultaneously, the absorbance of the sample solvent mixed with DPPH solution at a ratio of 1:3 (*v*/*v*) was measured and recorded as Ab. The DPPH clearance rate was calculated using Equation (2).

(2) $$P_{DPPH}=(1-\frac{A_{S}-A_{c}}{A_{b}})\times 100\%$$

In the formula: P_DPPH_—DPPH clearance rate,%; As—The absorbance of the sample mixed with DPPH solution at 517 nm; Ac—The absorbance of the sample mixed with its solvent at 517 nm; Ab—the absorbance of the DPPH solution mixed with the sample solvent at 517 nm.

Using Vc standard solution as the positive control, the Vc sample solution and DPPH solution were added together in a 2 mL centrifuge tube at a ratio of 1:3 (*v*/*v*). The mixture was left to react under light-free conditions at room temperature for 30 min. The reaction mixture was transferred to a 96-well plate, with three replicates per sample. The absorbance at 517 nm was measured and recorded as the Vc positive control value.

#### 3.8.2. ABTS Free Radical Scavenging Assay

A total of 200 mg of ABTS and 34.4 mg of K_2_(SO_4_)_2_ was dissolved in 50 mL of distilled water, and the mixture was allowed to stand overnight under light protection at room temperature to obtain an ABTS radical stock solution. Before use, it was diluted with anhydrous hydroethanolic and its absorbance at a wavelength of 734 nm was measured using a microplate reader (Lainde Intelligent Technology Co., Ltd., Shandong, China) to achieve a value of 0.7 ± 0.02, yielding the working solution. The herbal extract was mixed with ABTS working solution at a ratio of 1:9 (*v*/*v*), incubated under light protection at room temperature for 5 min, and the absorbance was measured [15] at a wavelength of 734 nm. The sample was replaced with its solvent and the absorbance was recorded as Ab. The ABTS clearance rate was calculated using Equation (3).

(3) $$P_{ABTS}=(\frac{A_{b}-A_{s}}{A_{b}})\times 100\%$$

In the formula: P_ABTS_—ABTS clearance rate,%; Ab—The absorbance of the ABTS working solution mixed with the sample solvent at 734 nm; As—The absorbance of the sample mixed with ABTS working solution at 734 nm.

Using the Vc standard as the positive control, the Vc sample solution and ABTS working solution were added in a 2 mL centrifuge tube at a ratio of 1:9 (*v*/*v*). The mixture was left to react for 5 min at room temperature away from light. The reaction mixture was transferred to a 96-well plate, with three replicates per sample. The absorbance at 734 nm was measured and recorded as the Vc positive control value.

### 3.9. Data Statistical Analysis

All experiments were conducted in triplicate (n = 3) unless otherwise specified, with results expressed as mean ± SD. The data analysis was performed using IBM SPSS Statistics 25 software. For multi-group comparisons, one-way ANOVA followed by Tukey’s post-hoc test was used, with exact *p*-values reported where *p* < 0.05. For comparisons between two groups, the Student’s *t*-test was applied. Statistical significance was defined at *p* < 0.05.

## 4. Conclusions

This study established a systematic “screening–combination–optimization–validation” framework to identify Folium *Aloe* and Cortex *Betulae* as the optimal synergistic drug pair from five traditional Chinese medicines. The combined extract exhibited enhanced antibacterial activity, with inhibition zones that increased by 21–36% compared to single-herb extracts and MIC values against *S. aureus* and *C. albicans* that reduced by 50% and 68.75%, respectively. Single-factor and Box–Behnken response surface optimization determined the optimal extraction parameters as a ratio of 1.53:1, a hydroethanolic concentration of 79.5%, and an extraction time of 2.6 h, with model R^2^ values of 0.9265–0.9783. The UPLC-QTOF-MS analysis identified nine major constituents, including anthraquinones, chromones, and triterpenoids, providing the phytochemical basis for the observed dual bioactivities. Mechanistic studies confirmed membrane disruption as the primary antibacterial mechanism through nucleic acid and protein leakage, whereas MBC/MIC ratios ≤3 demonstrated reliable bactericidal activity. The optimized extract achieved a DPPH scavenging rate of 90% at 1.0 mg/mL and ABTS activity comparable to ascorbic acid, with excellent cytocompatibility evidenced by >90% L929 cell viability even at 1000 μg/mL. Collectively, the Folium *Aloe*–Cortex *Betulae* pair realizes dual synergistic antibacterial and antioxidant effects through the shared redox chemistry of polyphenolic constituents, offering a robust experimental foundation for the development of functional wound dressings and green antibacterial biomaterials targeting chronic wound infections with oxidative stress microenvironments.

## Figures and Tables

**Table 1:** Response surface test results.

Run	X1	X2	X3	Y1	Y2	Y3	Y4
1	2	80	3	1.63333	1.76667	1.7	1.83333
2	1.5	70	3	1.71667	1.78333	1.71667	1.75
3	1.5	80	2.5	1.76667	1.88333	1.75	1.88333
4	1	80	3	1.68333	1.75	1.61667	1.76667
5	1.5	80	2.5	1.78333	1.86667	1.75	1.88333
6	2	80	2	1.66667	1.71667	1.68333	1.78333
7	1.5	80	2.5	1.76667	1.88333	1.73333	1.91667
8	1	80	2	1.65	1.75	1.65	1.75
9	2	90	2.5	1.7	1.75	1.7	1.76667
10	1.5	90	3	1.68333	1.81667	1.68333	1.8
11	2	70	2.5	1.71667	1.73333	1.71667	1.75
12	1.5	80	2.5	1.76667	1.88333	1.76667	1.88333
13	1.5	80	2.5	1.78333	1.88333	1.76667	1.9
14	1.5	90	2	1.68333	1.78333	1.7	1.81667
15	1	70	2.5	1.65	1.78333	1.58333	1.73333
16	1	90	2.5	1.66667	1.75	1.6	1.71667
17	1.5	70	2	1.7	1.85	1.65	1.7

**Table 2:** The identification of major chemical constituents in Folium Folium *Aloe* and Cortex *Betulae* extract by UPLC-QTOF-MS.

Title	RT (min)	Precursor *m*/*z*	PPM	Adduct	Fragment Ionse
Aloin	9.651383	419.1337	0.093049076	[M + H]^+^	239.0692; 211.07468; 257.07732
	9.617167	417.1202	2.615559864	[M − H]^−^	297.06805; 268.06748
Aloe-emodin	18.44568	269.0455	0.182125295	[M − H]^−^	269.03544; 241.04348; 227.0315; 225.04589
Betulin	22.52397	443.3881	0.577371951	[M + H]^+^	443.35057; 191.1786
Betulinic acid	21.15862	457.3676	0.045914925	[M + H]^+^	457.36207
Emodin	12.12848	269.0479	8.738297321	[M − H]^−^	269.04018; 227.03378
Aloeresin A	8.24265	541.1725	3.804716304	[M + H]^+^	541.17317; 147.04459; 275.09229; 395.13041
	8.6017	539.1522	6.842176957	[M − H]^−^	375.09844; 163.03801
Oleanolic acid	26.71362	455.3524	1.469189615	[M − H]^−^	455.34271; 437.32371; 411.33918; 409.327; 395.32094

**Table 3:** MIC and MBC Table.

mg/mL		*E. coli*	*S. aureus*	*T-Salmonella*	*C. albicans*
Folium *Aloe*	MIC	50	31.25	25	50
	MBC	100	62.5	50	125
Cortex *Betulae*	MIC	50	31.25	100	62.5
	MBC	125	75	200	125
Folium *Aloe* and Cortex *Betulae*	MIC	37.5	15.625	50	15.625
	MBC	62.5	25	75	31.25

**Table 4:** Single factor optimization experiment variables.

Factor	Level 1	Level 2	Level 3	Level 4	Level 5
Solvent concentration	pure water	20% hydroethanolic	40% hydroethanolic	60% hydroethanolic	80% hydroethanolic
Extraction time	1 h	1.5 h	2 h	2.5 h	3 h
Mixing ratio	2:1	1.5:1	1:1	1:1.5	1:2

**Table 5:** Factors and levels of the response surface test design.

Level	Factor		
	A: Mixing Ratio	B: Solvent Concentration	C: Extraction Time
−1	1:1	70% hydroethanolic	2 h
0	1:1.5	75% hydroethanolic	2.5 h
1	1:2	80% hydroethanolic	3 h

## Data Availability

The original contributions presented in this study are included in the article/Supplementary Material. Further inquiries can be directed to the corresponding author.

## Supplementary Materials

The following supporting information can be downloaded at: https://www.mdpi.com/article/10.3390/molecules31152590/s1, Figure S1: Original bacterial inhibition profiles of five traditional Chinese medicine aqueous extracts and hydroethanolic extracts; Figure S2: Original bacterial inhibition profiles of aqueous and hydroethanolic extracts from different drug combinations; Figure S3: Single-factor optimization plot for the combined extract of Folium *Aloe* and Cortex *Betulae*; Figure S4: Response surface optimization plot for the combined extract of Folium *Aloe* and Cortex *Betulae*; Figure S5: Response surface optimization contour plot of the combined extract from Folium *Aloe* and Cortex *Betulae*; Figure S6: MBC plots for Folium *Aloe*, Cortex *Betulae*, and their combined extract.
