# Supplementary material for: Synergistic Antibacterial and Antioxidant Activities of Folium Aloe–Cortex Betulae Drug Combination: Optimization via Box–Behnken Design and In Vitro Validation
Source: Molecules. 2026 Jul 24;31(15):2590. doi: 10.3390/molecules31152590 (PMC13468675; doi:10.3390/molecules31152590)
Supplement: Supplementary file 1 [file molecules-31-02590-s001.zip › molecules-4408671-supplementary.pdf]

Supplementary Materials

Synergistic Antibacterial and Antioxidant Activities of Folium *Aloe*–Cortex *Betulae* Drug Combination: Optimization via Box–Behnken Design and In Vitro Validation

Shun Zhang [1], Kun Bao [1], Yiyun Wei [1], Xu Liu [1] and Chenlu Zhang [1,2,3]*

1 School of Biological Science and Engineering, Shaanxi University of Technology, Hanzhong 723000, China; 15929874564@163.com (S.Z.); bkc0822@163.com (K.B.); 17764778099@163.com (Y.W.); 15667909234@163.com (X.L.)

2 Shaanxi Province Key Laboratory of Bio-Resources, Hanzhong 723000, China

3 Qinba State Key Laboratory of Biological Resources and Ecological Environment (Incubation), Hanzhong 723000, China

* Correspondence: chenluzhang@snut.edu.cn; Tel.: +86-158-9166-2100

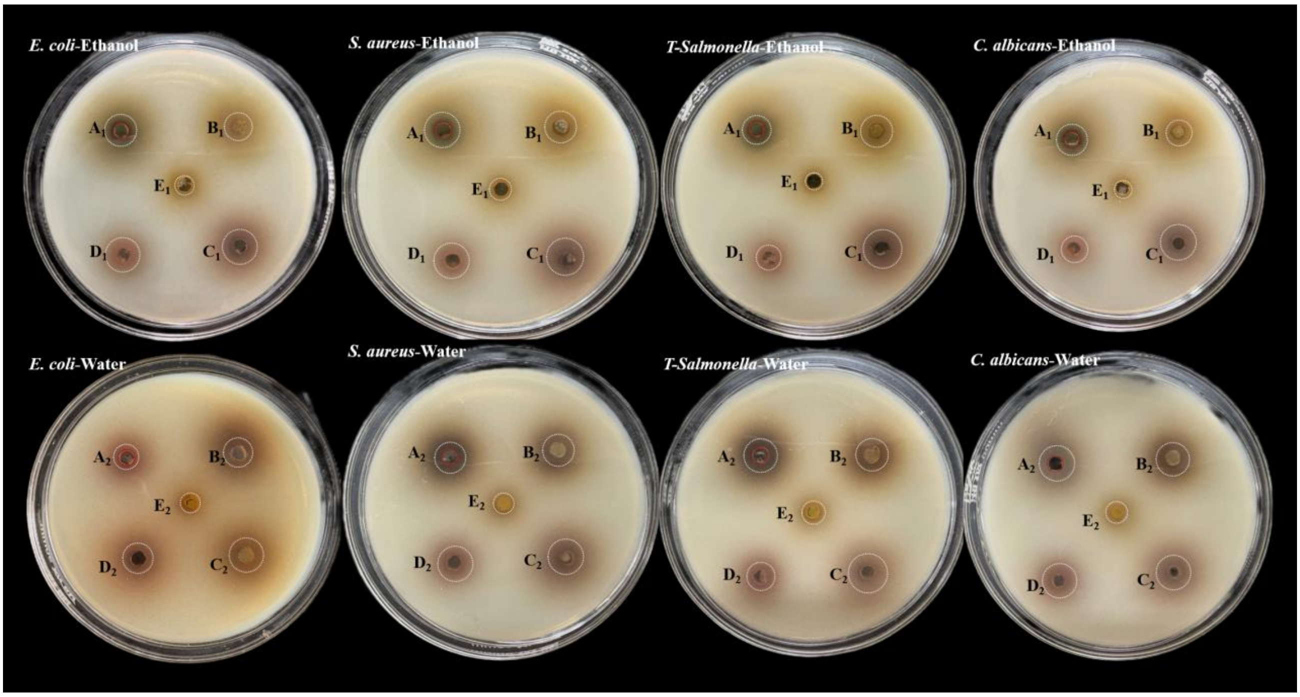

Figure S1: **Original bacterial inhibition profiles of five traditional Chinese medicine aqueous extracts and alcoholic extracts**

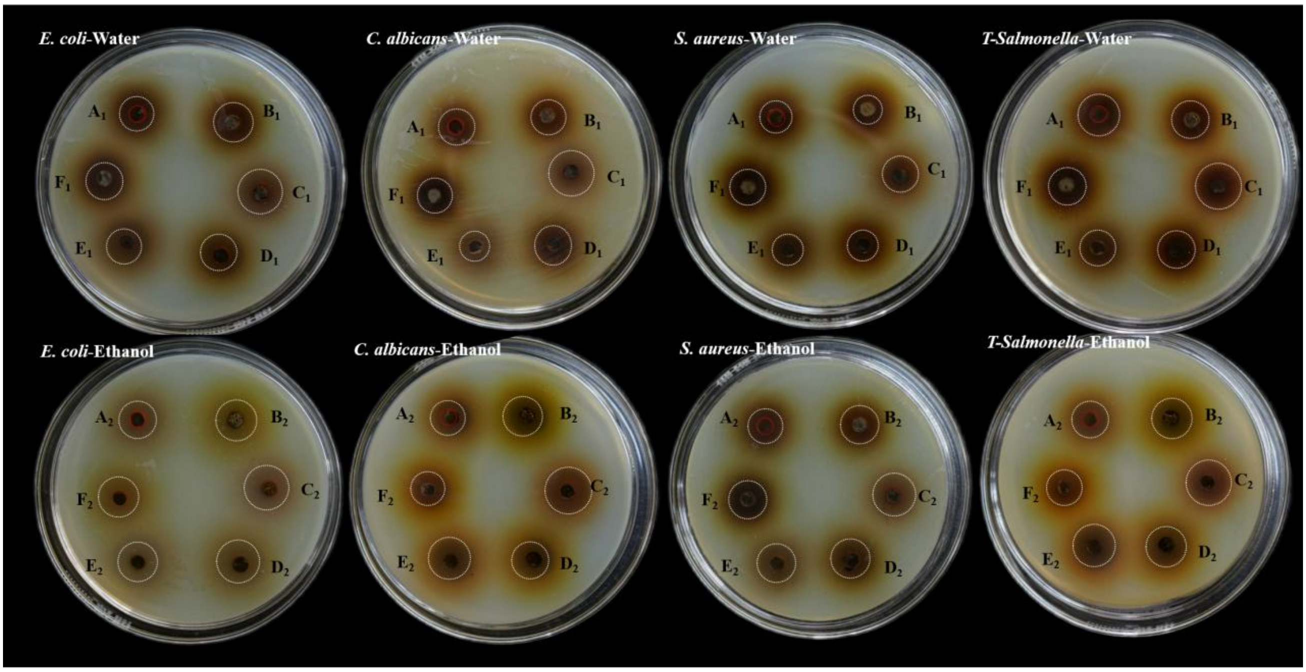

Figure S2: **Original bacterial inhibition profiles of aqueous and alcoholic extracts from different drug combinations**

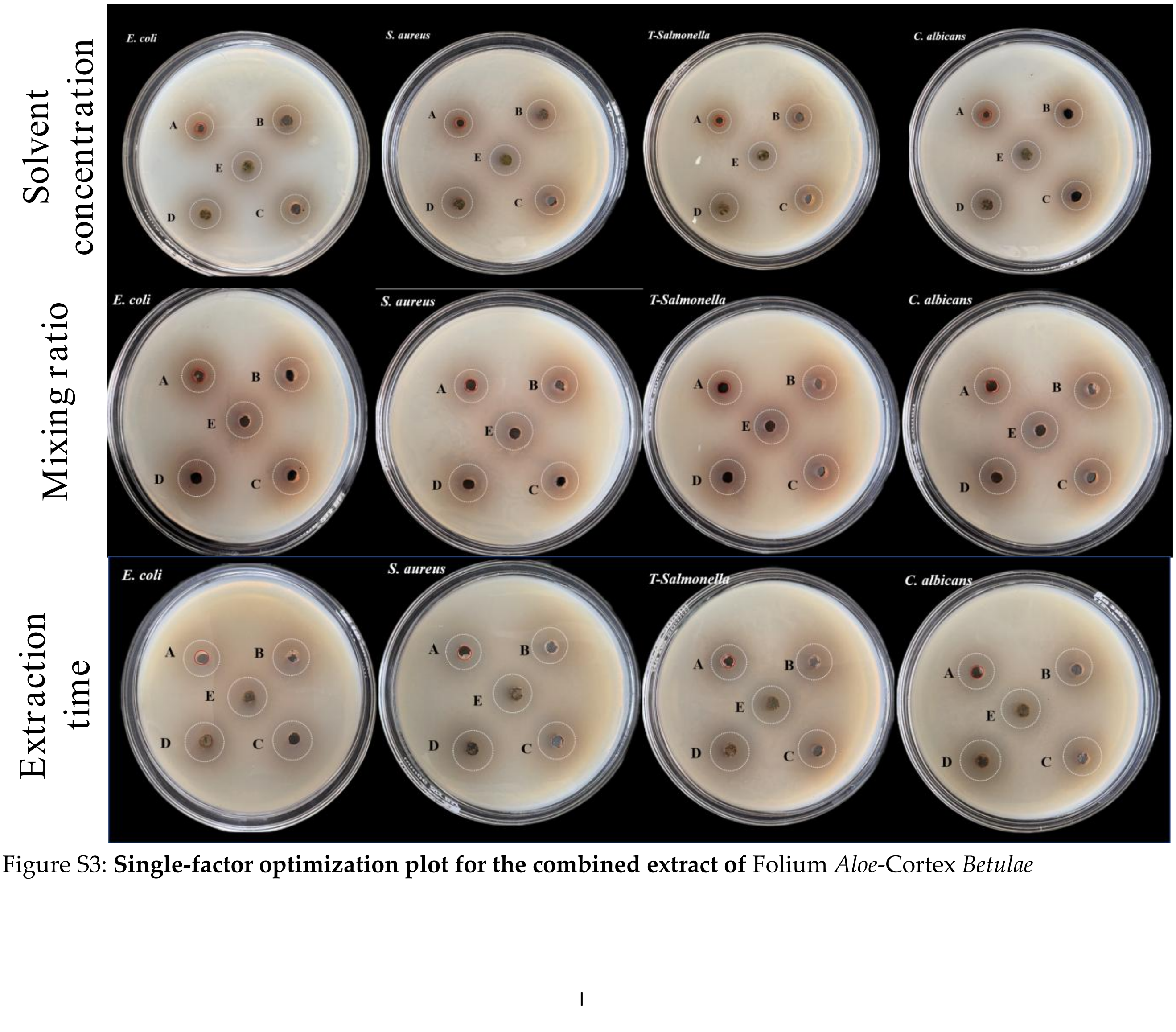

Figure S3: **Single-factor optimization plot for the combined extract of** Folium *Aloe*-Cortex *Betulae*

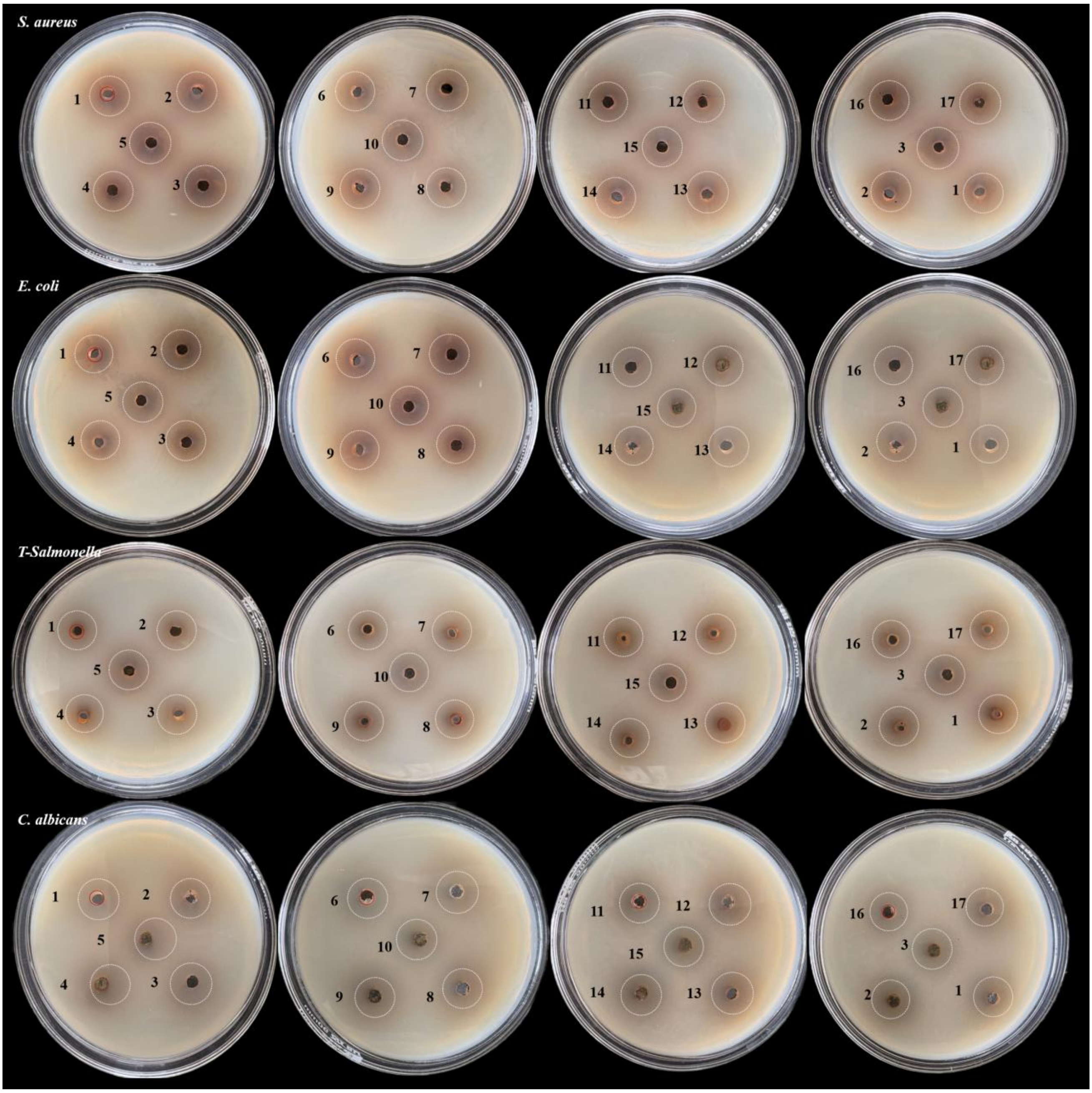

Figure S4: **Response surface optimization plot for the combined extract of** Folium *Aloe*-Cortex *Betulae*

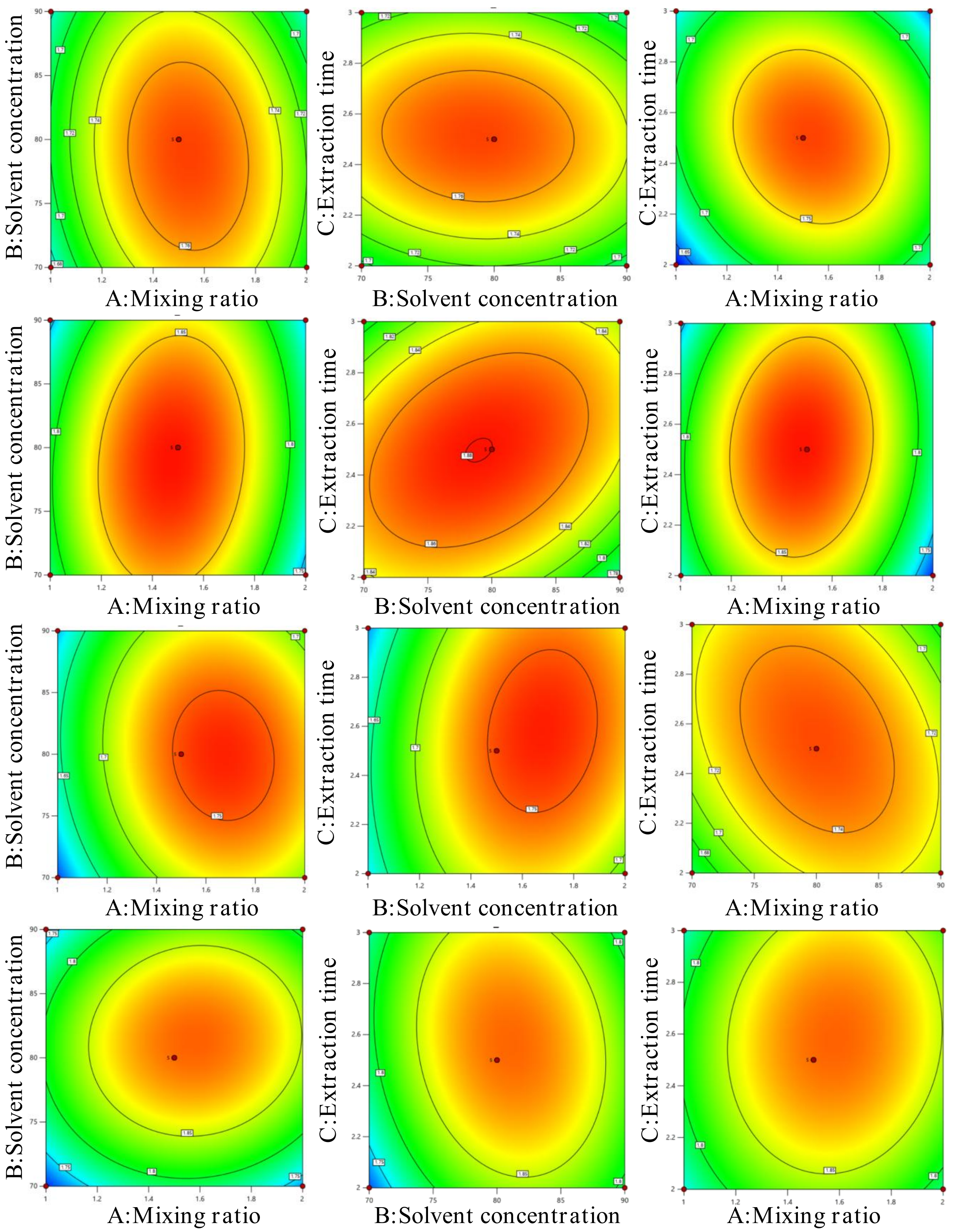

Figure S5: **Response surface optimization contour plot of the combined extract from** Folium *Aloe*-Cortex *Betulae*

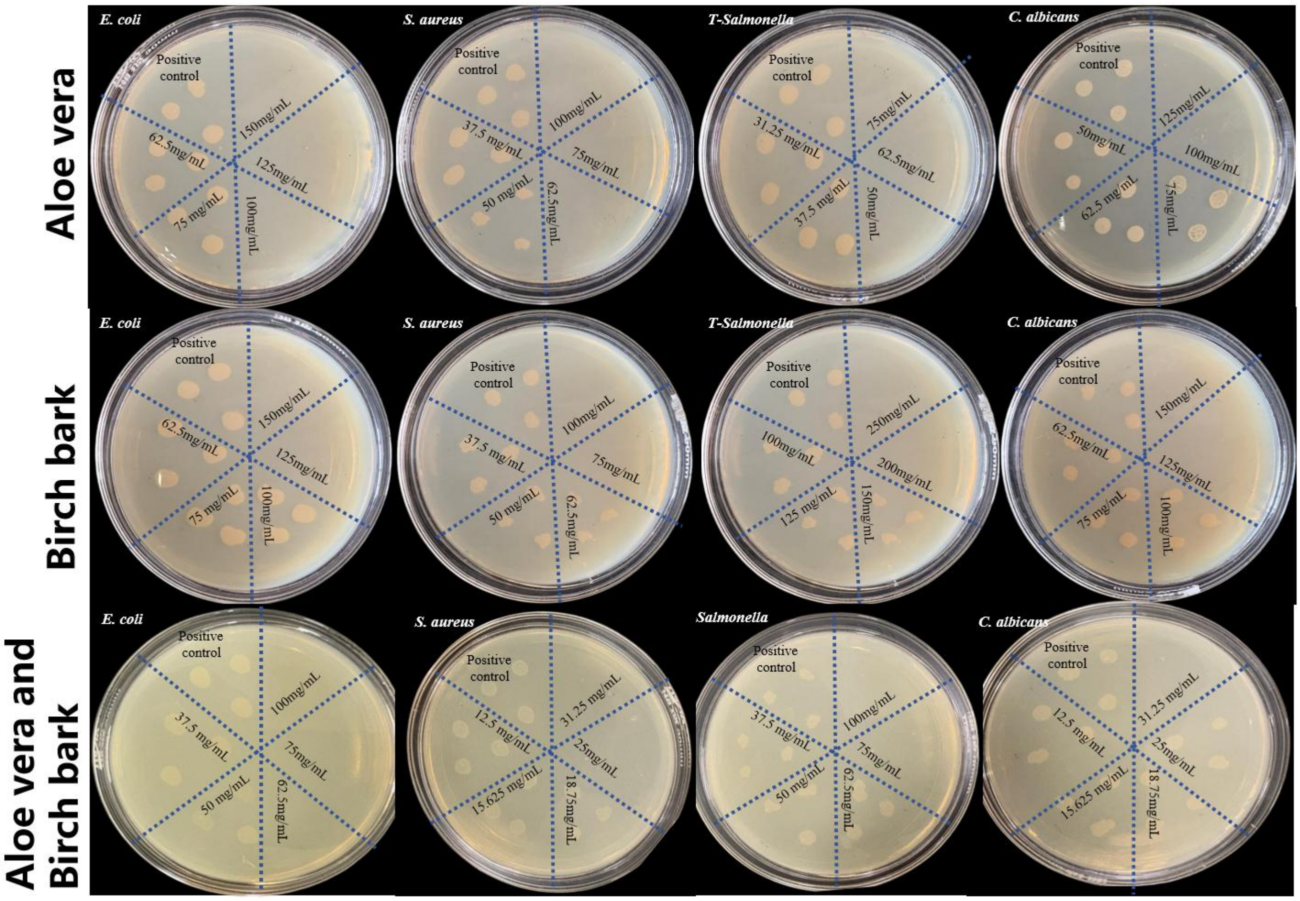

Figure S6: MBC plots for Folium *Aloe,* Cortex *Betulae,* and their combined extract
